# Obtaining a Proportional Allocation by Deleting Items

**DOI:** 10.1007/s00453-020-00794-4

**Published:** 2021-03-26

**Authors:** Britta Dorn, Ronald de Haan, Ildikó Schlotter

**Affiliations:** 1 University of Tübingen, Tübingen, Germany; 2 ILLC, University of Amsterdam, Amsterdam, the Netherlands; 3 Budapest University of Technology and Economics, and Centre for Economic and Regional Studies, Institute of Economics, Budapest, Hungary

**Keywords:** Fair division, Proportional allocation, Control, Item deletion, Computational complexity, Parameterized complexity

## Abstract

We consider the following control problem on fair allocation of indivisible goods. Given a set *I* of items and a set of agents, each having strict linear preferences over the items, we ask for a minimum subset of the items whose deletion guarantees the existence of a proportional allocation in the remaining instance; we call this problem Proportionality by Item Deletion (PID). Our main result is a polynomial-time algorithm that solves PID for three agents. By contrast, we prove that PID is computationally intractable when the number of agents is unbounded, even if the number *k* of item deletions allowed is small—we show that the problem is ${\mathsf {W}}[3]$-hard with respect to the parameter *k*. Additionally, we provide some tight lower and upper bounds on the complexity of PID when regarded as a function of |*I*| and *k*. Considering the possibilities for approximation, we prove a strong inapproximability result for PID. Finally, we also study a variant of the problem where we are given an allocation $\pi$ in advance as part of the input, and our aim is to delete a minimum number of items such that $\pi$ is proportional in the remainder; this variant turns out to be ${{\mathsf {N}}}{{\mathsf {P}}}$-hard for six agents, but polynomial-time solvable for two agents, and we show that it is $\mathsf {W[2]}$-hard when parameterized by the number *k* of

## Introduction

We consider a situation where a set *I* of indivisible items needs to be allocated to a set *N* of agents in a way that is perceived as *fair*. Unfortunately, it may happen that a fair allocation does not exist in a setting. In such situations, we might be interested in the question how our instance can be modified in order to achieve a fair outcome. Naturally, we seek for a modification that is as small as possible. This can be thought of as a *control action* carried out by a central agency whose task is to find a fair allocation. The computational study of such control problems was first proposed by Bartholdi, III et al. [5] for voting systems; our paper follows the work of Aziz et al. [4] who have recently initiated the systematic study of control problems in the area of fair division.

The idea of fairness can be formalized in various different ways such as proportionality, envy-freeness, or max-min fair share (see the book chapter by Bouveret et al. [6] for an introduction). Here we focus on *proportionality*, a notion originally defined in a model where agents use utility functions to represent their preferences over items. In that context, an allocation is called proportional if each agent obtains a set of items for which their utility is at least 1/|*N*| of their total utility of all items. One way to adapt this notion to a model with linear preferences (not using explicit utilities) is to look for an allocation that is proportional with respect to *any* choice of utility functions for the agents that is compatible with the given linear preferences. Aziz et al. [3] referred to this property as “necessary proportionality”; for simplicity, we use the shorter term “proportionality.” For a survey of other possible notions of proportionality and fairness under linear preferences, we also refer to Aziz et al. [3].

We have several reasons for considering linear preferences. First, the most important advantage of this setting is the easier elicitation of agents’ preferences. In many practical applications, especially with a large number of items, it is unrealistic to assume that agents are able to assign a meaningful cardinal value to each of the items. This may be due to lack of information, e.g., when agents need to declare preferences over items about which they have incomplete knowledge, or an unwillingness to associate a determined value for each item: in scenarios where the usefulness or virtue of an item cannot be simply measured by its monetary value (e.g., students ranking assignments, shared owners of a holiday home ranking time slots, heirs ranking family assets), people may find it much more convenient to express their preferences in an ordinal way, thus reducing their cognitive burden. Besides easier elicitation, it is important to note that it is also easier to visualize ordinal preferences than cardinal ones, which may have significance when we wish to elicit preferences from children or people with impaired cognitive abilities. Hence, ordinal preferences may be more useful in practical applications. From a technical point of view, this simpler model is more tractable in a computational sense: under linear preferences, the existence of a proportional allocation can be decided in polynomial time [3], whereas the same question for cardinal utilities is NP-hard [20]. Since Lipton et al. [20] show the NP-hardness of the problem already for two agents, we do not even have hope for an FPT-algorithm with the number of agents as the parameter. Clearly, if already the existence of a proportional allocation is computationally hard to decide, then we have no hope to solve the corresponding control problem efficiently.

Control actions can take various forms. Aziz et al. [4] mention several possibilities: control by adding/deleting/replacing agents or items in the given instance, or by partitioning the set of agents or items. In this paper we concentrate only on control by *item deletion*, where the task is to find a subset of the items, as small as possible, whose removal from the instance guarantees the existence of a proportional allocation. In other words, we ask for the maximum number of items that can be allocated to the agents in a proportional way.

### Related Work

We follow the research direction proposed by Aziz et al. [4] who initiated the systematic study of control problems in the area of fair division. As an example, Aziz et al. [4] consider the complexity of obtaining envy-freeness by adding or deleting items or agents, assuming linear preferences. They show that adding/deleting a minimum number of items to ensure envy-freeness can be done in polynomial time for two agents, while for three agents it is NP-hard even to decide if an envy-free allocation exists. As a consequence, they obtain NP-hardness also for the control problems where we want to ensure envy-freeness by adding/deleting items in case there are more than two agents, or by adding/deleting agents.

The problem of deleting a minimum number of items to obtain envy-freeness was first studied by Brams et al. [7] who gave a polynomial-time algorithm for the case of two agents.^1^

In a setting with cardinal utilities, Caragiannis et al. [8] propose a model where items can be donated (i.e., deleted) before allocating the rest to agents; they propose an algorithm that, after deleting a set of items, yields an allocation for the remaining items that is envy-free up to any goods, and whose Nash welfare value is at least half of the optimum. In the context of cake cutting, Segal-Halevi et al. [24] proposed the idea of distributing only a portion of the entire cake in order to obtain an envy-free allocation efficiently.

Looking at the topic in a broader sense, several papers have investigated possible ways to achieve fairness by certain types of control actions. A prominent example is hiding information from agents in order to facilitate a fair allocation. Chen and Shah [10] have found that if agents do not receive any information about the items allocated to others, then the expected amount of envy experienced by the agents typically reduces. Aziz et al. [2] investigated a model where the information that agents obtain on the allocation is based on a graph representing social contacts. Hosseini et al. [18] have proposed an algorithm that eliminates envy through withholding information about a set of few items. Halpern and Shah [16] have also examined the possibilities for overcoming envy by subsidies where agents receive monetary compensation.

For the Hospitals/Residents with Couples problem, Nguyen and Vohra [22] considered yet another type of control action: they obtained stability by slightly perturbing the capacities of hospitals.

### Our Contribution

We first consider the case where the number of agents is unbounded (see Sect. 3). We show that the problem of deciding whether there exist at most *k* items whose deletion allows for a proportional allocation is NP-complete, and that this problem is ${\mathsf {W}}[3]$-hard with parameter *k* (see Theorem 2). This latter result shows that even if we allow only a few items to be deleted, we cannot expect an efficient algorithm, since the problem is not fixed-parameter tractable with respect to the parameter *k* (unless $\mathsf {FPT} = {\mathsf {W}}[3]$, which is widely believed not to be the case).

Additionally, we provide tight upper and lower bounds on the complexity of the problem. In Theorem 3 we prove that the trivial $|I|^{O(k)}$ time algorithm—that, in a brute force manner, checks for each subset of *I* of size at most *k* whether it is a solution—is essentially optimal (under the widely accepted assumption that $\mathsf {FPT} \ne {\mathsf {W}}[1]$). We provide another simple algorithm in Theorem 4 that has optimal running time, assuming the Exponential Time Hypothesis.

Next, we look at the possibilities of approximation in Sect. 3.1. First we focus on the approximation problem where the objective is to minimize the number *k* of item deletions, and we provide a strong inapproximability result in Theorem 5 by proving that not even an FPT-algorithm with parameter *k* can yield a ratio of $|I|^{1-\varepsilon }$ for some constant $\varepsilon >0$. Next, we examine the possibilities for maximizing the number of items that agents obtain under a proportional allocation. In Corollary 1, we observe that it is ${{\mathsf {N}}}{{\mathsf {P}}}$-hard to decide if there exists a set of 2|*N*| items which can be allocated to our set *N* of agents in a proportional way. Contrasting this result, we propose a simple polynomial-time algorithm in Theorem 6 that allocates one item to each agent proportionally, whenever this is possible.

In Section 4, we turn our attention to the case with only three agents. In Theorem 7 we propose a polynomial-time algorithm for this case, which can be viewed as our main result. The presented algorithm is based on dynamic programming, but relies heavily on a non-trivial insight into the structure of solutions.

Finally, in Sect. 5 we briefly look at the variant of our problem where we are given a fixed allocation in advance, and the task is to decide whether we can make the given allocation proportional by deleting certain items. We prove that this problem is easy for two agents (Theorem 8), but becomes ${{\mathsf {N}}}{{\mathsf {P}}}$-hard for six agents (Theorem 9). The computational intractability persists even if the number of deletions is small, as evidenced by Theorem 10 that proves ${\mathsf {W}}[2]$-hardness with parameter *k* for this variant.

## Preliminaries and Definitions

In this section, we revisit some technical concepts and notions that we use in the remainder of the paper. We also give a formal definition of the problem of Proportionality by Item Deletion (PID).

**(Parameterized) complexity theory** We assume the reader to be familiar with basic notions from the theory of computational complexity—in particular, with the complexity classes ${\mathsf {P}}$ and ${{\mathsf {N}}}{{\mathsf {P}}}$, with the notion of polynomial-time algorithms and polynomial-time (many-to-one) reductions, and with the notion of ${{\mathsf {N}}}{{\mathsf {P}}}$-hardness and -completeness. For more details, we refer to textbooks on the topic (see, e.g., the book by Arora and Barak [1]).

We review some basic notions from parameterized complexity theory—for more details, see, e.g., the book by Downey and Fellows [13]. In parameterized complexity theory, in addition to the size |*x*| of the input *x* of a problem, one considers a problem parameter *k*. This parameter is intended to measure some type of structure that is present in the input. The aim then is to obtain *fixed-parameter tractable algorithms* (or *FPT-algorithms*), that have running time $f(k) \cdot |x|^{O(1)}$, for some computable function *f*. This is in contrast with the central notion of tractable algorithms in classical complexity theory: polynomial-time algorithms, i.e., algorithms running in time $|x|^{O(1)}$. The class of all parameterized problems that admit an FPT-algorithm is denoted by $\mathsf {FPT}$.

In addition to the class $\mathsf {FPT}$, parameterized complexity theory features *parameterized intractability classes*. These are classes containing problems that are considered to be unlikely to have fixed-parameter tractable algorithms. The most prominent examples of such classes are the classes ${\mathsf {W}}[t]$ for $t\in \{1,2,3,\dots \}$. For a formal definition of these classes, we refer to textbooks (e.g., [13] or [15]). It holds that ${\mathsf {W}}[1] \subseteq {\mathsf {W}}[2] \subseteq {\mathsf {W}}[3]$, and it is widely believed that $\mathsf {FPT} \ne {\mathsf {W}}[1]$.

To give evidence that a problem is not fixed-parameter tractable, one typically uses *FPT-reductions* to show that the problem is ${\mathsf {W}}[t]$*-hard* for some *t*. If the problem admitted an FPT-algorithm, then this would imply that $\mathsf {FPT} = {\mathsf {W}}[t]$—in other words, under the assumption that $\mathsf {FPT} \ne {\mathsf {W}}[t]$, the problem is not fixed-parameter tractable. An *FPT-reduction* from a parameterized problem $Q_1$ to a parameterized problem $Q_2$ is a function *R* that takes an input (*x*, *k*) of $Q_1$, and produces an input $(x',k')$ of $Q_2$, such that: (i) $(x,k) \in Q_1$ if and only if $(x',k') \in Q_2$; (ii) runs in time $f(k) \cdot |x|^{O(1)}$, for some computable function *f*; and (iii) $k' \le g(k)$, for some fixed computable function *g*. A problem *Q* is ${\mathsf {W}}[t]$-hard for some *t*, if every problem in ${\mathsf {W}}[t]$ can be FPT-reduced to *Q*.

**Preferences** Let *N* be a set of agents and *I* a set of indivisible items that we wish to allocate to the agents in some way. We assume that each agent $a \in N$ has strict preferences over the items, expressed by a preference list $L^a$ that is a linear ordering of *I*, and we set $L=\{L^a\mid a \in N\}$. We call the triple (*N*, *I*, *L*) a *(preference) profile*. We denote by $L^a[i:j]$ the subsequence of $L^a$ containing the items ranked by agent *a* between the positions *i* and *j*, inclusively, for any $1 \le i \le j \le |I|$. Also, for a subset $X \subseteq I$ of items we denote by $L^a_X$ the restriction of $L^a$ to the items in *X*.

**Proportionality** Interestingly, Pruhs and Woeginger [23, Lemma 1] gave an equivalent definition for the concept of proportionality (as described in Sect. 1) that is more direct and practical: we say that an allocation $\pi : I \rightarrow N$ mapping items to agents is *proportional* if for any integer $i \in \{1, \dots , |I|\}$ and any agent $a \in N$, the number of items from $L^a[1:i]$ allocated to *a* by $\pi$ is at least *i*/|*N*|. Note that, in particular, this means that in a proportional allocation, each agent needs to get his or her first choice. Another important observation is that a proportional allocation can only exist if the number of items is a multiple of |*N*|, since each agent needs to obtain at least |*I*|/|*N*| items.

**Control by deleting items** Given a profile ${\mathcal {P}}=(N,I,L)$ and a subset *U* of items, we can define the preference profile ${\mathcal {P}}-U$ obtained by removing all items in *U* from *I* and from all preference lists in *L*. Let us define the Proportionality by Item Deletion (PID) problem as follows. The input of PID is a pair $({\mathcal {P}},k)$ where ${\mathcal {P}}=(N,I,L)$ is a preference profile and *k* is an integer. We call a set $U \subseteq I$ of items a *solution* for ${\mathcal {P}}$ if its removal from *I* allows for proportionality, that is, if there exists a proportional allocation $\pi :I \setminus U \rightarrow N$ for ${\mathcal {P}}-U$. The task in PID is to decide if there exists a solution for ${\mathcal {P}}$ of size at most *k*. Note that the number of items remaining after the removal of the solution must be a multiple of |*N*|.

## Unbounded Number of Agents

The existence of a proportional allocation can be decided in polynomial time by checking whether a certain bipartite graph corresponding to our instance admits a perfect matching [23, Lemma 4]. Therefore the Proportional Item Deletion problem is solvable in $|I|^{O(k)}$ time by the brute force algorithm that checks for each subset of *I* of size at most *k* whether it is a solution. In terms of parameterized complexity, this means that PID parameterized by the solution size *k* is in ${{\mathsf {X}}}{{\mathsf {P}}}$, i.e., the class of parameterized problems that can be solved in polynomial time for any constant value of the parameter.

Clearly, such a brute force approach may only be feasible if the number *k* of items we are allowed to delete is very small. Searching for a more efficient algorithm, one might ask whether the problem becomes fixed-parameter tractable with *k* as the parameter, i.e., whether there exists an algorithm for PID that, for an instance $({\mathcal {P}},k)$ runs in time $f(k) |{\mathcal {P}}|^{O(1)}$ for some computable function *f*. Such an algorithm could be much faster in practice compared to the brute force approach described above.

Unfortunately, the next theorem shows that finding such a fixed-parameter tractable algorithm seems unlikely, as PID is ${\mathsf {W}}[2]$-hard with parameter *k*. Hence, deciding whether the deletion of *k* items can result in a profile admitting a proportional allocation is computationally intractable even for small values of *k*. (After showing ${\mathsf {W}}[2]$-hardness, we will show that this result can in fact be strengthened to ${\mathsf {W}}[3]$-hardness. We present the ${\mathsf {W}}[2]$-hardness result for two reasons: (1) it is conceptually simpler than the ${\mathsf {W}}[3]$-hardness proof; and (2) its proof will be useful for showing other lower bounds—namely, Theorems 3 and 5.)

### Theorem 1

Proportionality by Item Deletion is NP-complete and ${\mathsf {W}}[2]$-hard when parameterized by the size *k* of the desired solution.

### Proof

We are going to present an FPT-reduction from the ${\mathsf {W}}[2]$-hard problem *k* -Dominating Set, where we are given a graph $G=(V,E)$ and an integer *k*, and the task is to decide if *G* contains a dominating set of size at most *k*; a vertex set $D \subseteq V$ is *dominating* in *G* if each vertex in *V* is either in *D* or has a neighbor in *D*. We denote by *N*(*v*) the set of neighbors of some vertex $v \in V$, and we let $N[v]=N(v) \cup \{v\}$. Thus, a vertex set *D* is dominating if $N[v] \cap D \ne \emptyset$ holds for each $v \in V$.

Let us construct an instance $I_{PID }=({\mathcal {P}},k)$ of PID with ${\mathcal {P}}=(N,I,L)$ as follows. We let *N* contain $3n+2m+1$ agents where $n=|V|$ and $m=|E|$: we create $n+1$ so-called *selection agents* $s_1, \dots , s_{n+1}$, and for each $v \in V$ we create a set $A_v=\{a_v^j \mid 1 \le j \le |N[v]|+1\}$ of *vertex agents*. Next we let *I* contain $2|N|+k$ items: we create distinct first-choice items *f*(*a*) for each agent $a \in N$, a *vertex item* $i_v$ for each $v \in V$, a dummy item $d_v^j$ for each vertex agent $a_v^j \in N$, and $k+1$ additional dummy items $c_1, \dots , c_{k+1}$.

Let *F* denote the set of all first-choice items, i.e., $F=\{f(a) \mid a \in N\}$. For any set $U \subseteq V$ of vertices in *G*, let $I_U=\{i_v \mid v \in U\}$; in particular, $I_V$ denotes the set of all vertex items.

Before defining the preferences of agents, we need some additional notation. We fix an arbitrary ordering $\prec$ over the items, and for any set *X* of items we let [*X*] denote the ordering of *X* according to $\prec$. Also, for any $a \in N$, we define the set $F^a_i$ to contain the first *i* elements of $[ F \setminus \{f(a)\} ]$, for any $i \in \{1, \dots , |N|-1\}$. We end preference lists below with the symbol ‘’ meaning all remaining items not listed explicitly, ordered according to $\prec$.

Now we are ready to define the preference list $L^a$ for each agent *a*.

- If *a* is a selection agent $a=s_i$ with $1 \le i \le n-k$, then let
- If *a* is a selection agent $a=s_i$ with $n-k < i \le n+1$, then let
- If *a* is a vertex agent $a=a_v^j$ for some $v \in V$ with $1 \le j \le |N[v]|+1$, then let

This finishes the definition of our PID instance $I_{PID }$.

Suppose that there exists a solution *S* of size at most *k* to $I_{PID }$ and a proportional allocation $\pi$ mapping the items of $I \setminus S$ to the agents in *N*. Observe that by $|I|=2|N|+k$, we know that *S* must contain exactly *k* items.

First, we show that *S* cannot contain any item from *F*. For contradiction, assume that $f(a) \in S$ for some agent *a*. Since the preference list of *a* starts with more than *k* items from *F* (by $N-n>n>k$), the first item in $L^a_{I \setminus S}$ must be an item *f*(*b*) for some $b \in N$, $b \ne a$. The first item in $L^b_{I \setminus S}$ is exactly *f*(*b*), and thus any proportional allocation should allocate *f*(*b*) to both *a* and *b*, a contradiction.

Next, we prove that $S \subseteq I_V$. For contradiction, assume that *S* contains less than *k* items from $I_V$. Then, after the removal of *S*, the top $|N|+1$ items in the preference list $L^{s_i}_{I \setminus S}$ of any selection agent $s_i$ are all contained in $I_V \cup F$. Hence, $\pi$ must allocate at least two items from $I_V \cup F$ to $s_i$, by the definition of proportionality. Recall that for any agent *a*, $\pi$ allocates *f*(*a*) to *a*, meaning that $\pi$ would need to distribute the *n* items in $I_V$ among the $n+1$ selection agents, a contradiction. Hence, we have $S \subseteq I_V$.

We claim that the *k* vertices $D=\{v \mid i_v \in S\}$ form a dominating set in *G*. Let us fix a vertex $v \in V$. For sake of contradiction, assume that $N[v] \cap D =\emptyset$, and consider any vertex agent *a* in $A_v$. Then the top $|N|+1$ items in $L^a_{I \setminus S}$ are the same as the top $|N|+1$ items in $L^a=L^a_I$ (using that $S \cap F=\emptyset$), and these items form a subset of $I_{N[v]} \cup F$ for every $a \in A_v$. But then arguing as above, we get that $\pi$ would need to allocate an item of $I_{N[v]}$ to each of the $|N[v]|+1$ vertex agents in $A_v$; again a contradiction. Hence, we get that $N[v] \cap D \ne \emptyset$ for each $v \in V$, showing that *D* is indeed a dominating set of size *k*.

For the other direction, let *D* be a dominating set of size *k* in *G*, and let *S* denote the set of *k* vertex items $\{i_v \mid v \in D\}$. To prove that *S* is a solution for $I_{PID }$, we define a proportional allocation $\pi$ in the instance obtained by removing *S*. First, for each selection agent $s_i$ with $1 \le i \le n-k$, we let $\pi$ allocate $f(s_i)$ and the *i*th item from $I_{V \setminus D}$ to $s_i$ . Second, for each selection agent $s_{n-k+i}$ with $1 \le i \le k+1$, we let $\pi$ allocate $f(s_{n-k+i})$ and the dummy item $c_i$ to $s_{n-k+i}$. Third, $\pi$ allocates the items $f(a_v^j)$ and $d_v^j$ to each vertex agent $a_v^j \in N$.

It is straightforward to check that $\pi$ is indeed proportional.

For proving ${{\mathsf {N}}}{{\mathsf {P}}}$-completeness, observe that the presented FPT-reduction is a polynomial-time reduction as well, so the ${{\mathsf {N}}}{{\mathsf {P}}}$-hardness of Dominating Set implies that PID is ${{\mathsf {N}}}{{\mathsf {P}}}$-hard as well; since for any subset of the items we can verify in polynomial time whether it yields a solution, containment in ${{\mathsf {N}}}{{\mathsf {P}}}$ follows. $\square$

As mentioned above, we can in fact strengthen the ${\mathsf {W}}[2]$-hardness result of Theorem 1 and show that PID is even ${\mathsf {W}}[3]$-hard with respect to parameter *k*.

### Theorem 2

Proportionality by Item Deletion is ${\mathsf {W}}[3]$-hard when parameterized by the size *k* of the desired solution.

### Proof

We are going to present an FPT-reduction from the ${\mathsf {W}}[3]$-hard wcs$^-$[3] problem, which is the weighted satisfiability problem for formulas of the form $\varphi = \bigwedge \nolimits _{i=1}^{m_1} \bigvee \nolimits _{j=1}^{m_{2,i}} \bigwedge \nolimits _{\ell =1}^{m_{3,i,j}} l_{i,j,\ell }$, where each $l_{i,j,\ell }$ is a negative literal [14, Theorem 4.13] (see also [11, 15]). Let $(\varphi ,k)$ be an instance of the weighted satisfiability problem, where $\varphi$ is a formula of the form described above; the task in wcs$^-$[3] is to decide whether there is a truth assignment of weight *k* that satisfies $\varphi$. Let $X = \{ x_1,\dotsc ,x_n \}$ be the set of variables occurring in $\varphi$— that is, *n* denotes the number of variables in $\varphi$. We will construct an instance $I_{PID }=({\mathcal {P}},k)$ of PID with ${\mathcal {P}}=(N,I,L)$ as follows. We let *N* contain $n+1+\sum \nolimits _{i = 1}^{m_1} m_{2,i}$ agents: we create $n+1$ so-called *selection agents* $s_1,\dotsc ,s_{n+1}$, and for each $1 \le i \le m_1$ we create a set $A_i =\{a_i^j \mid 1 \le j \le m_{2,i}\}$ of *verification agents*. Next we let *I* contain $2|N|+k$ items: we create distinct *first-choice items* *f*(*a*) for each agent $a \in N$, a *variable item* $w_u$ for each $1 \le u \le n$, $m_{2,i}$ *verification items* $y_{i,1},\dotsc ,y_{i,m_{2,i}}$ for each $1 \le i \le m_1$, and $k+1$ *dummy items* $c_1, \dots , c_{k+1}$.

Let *F* denote the set of all first-choice items, i.e., $F=\{f(a) \mid a \in N\}$. For any subset $X' \subseteq X$ of variables, let $W_{X'}=\{w_u \mid x_u \in X' \}$; in particular, $W_X$ denotes the set of all variable items.

Before defining the preferences of agents, we need the additional notation used also in the proof of Theorem 1. We fix an arbitrary ordering $\prec$ over the items, and for any set *Z* of items we let [*Z*] denote the ordering of *Z* according to $\prec$. Also, for any $a \in N$, we define the set $F^a_i$ to contain the first *i* elements of $[F \setminus \{f(a)\}]$, for any $i \in \{1, \dots , |N|-1\}$. Moreover, for any $1 \le i \le m_1$ we define the sets $Y_i = \{ y_{i,1},\dotsc ,y_{i,m_{2,i}} \}$ and $Y'_i = \{ y_{i,1},\dotsc ,y_{i,m_{2,i}-1} \}$. We end preference lists below with the symbol ‘’ meaning all remaining items not listed explicitly, ordered according to $\prec$.

Now we are ready to define the preference list $L^a$ for each agent *a*.

- If *a* is a selection agent $a=s_i$ with $1 \le i \le n-k$, then let
- If *a* is a selection agent $a=s_i$ with $n-k < i \le n+1$, then let
- If *a* is a verification agent $a=a_i^j$ for $1 \le i \le m_1$ and $1 \le j \le m_{2,i}$, then let  where $C_{i,j} = X \setminus \{ x \in X \mid l_{i,j,\ell } = \lnot x \text { for some } 1 \le \ell \le m_{3,i,j}\}$ is the set of variables that do not occur in any literal $l_{i,j,\ell }$, for $1 \le \ell \le m_{3,i,j}$.

This finishes the definition of our PID instance $I_{PID }$.

Suppose that there exists a solution *S* of size at most *k* to $I_{PID }$ and a proportional allocation $\pi$ mapping the items of $I \setminus S$ to the agents in *N*. Observe that by $|I|=2|N|+k$, we know that *S* must contain exactly *k* items.

First, we show that *S* cannot contain any item from *F*. To derive a contradiction, assume that $f(a) \in S$ for some agent *a*. We can safely assume that $|N|-n>k$ and that $|N| - n > m_{2,i}$ for each $1 \le i \le m_1$. As a result, the preference list of *a* starts with more than *k* items from *F*. Therefore, the first item in $L^a_{I \setminus S}$ must be an item *f*(*b*) for some $b \in N$, $b \ne a$. Clearly, the first item in $L^b_{I \setminus S}$ is exactly *f*(*b*), which means that any proportional allocation should allocate *f*(*b*) to both *a* and *b*, which is a contradiction.

Next, we prove that $S \subseteq W_X$. To derive a contradiction, assume that *S* contains less than *k* items from $W_X$. Then, after the removal of *S*, the top $|N|+1$ items in the preference list $L^{s_i}_{I \setminus S}$ of any selection agent $s_i$ are all contained in $W_X \cup F$. Hence, $\pi$ must allocate at least two items from $W_X \cup F$ to each $s_i$, by the definition of proportionality. Recall that for any agent *a*, $\pi$ allocates *f*(*a*) to *a*, meaning that $\pi$ would need to distribute the *n* items in $W_X$ among the $n+1$ selection agents, which is a contradiction. Hence, we have $S \subseteq W_X$. We also get that $\pi$ must allocate all items in $W_X \setminus S \cup \{c_1, \dots , c_{k+1}\}$ to the selection agents.

Consider the truth assignment $\alpha : X \rightarrow \{ 0,1 \}$ defined by letting $\alpha (x_u) = 1$ if and only if $w_u \in S$, for each $x_u \in X$. Since $|S| = k$, the truth assignment $\alpha$ has weight *k*. We show that $\alpha$ satisfies $\varphi$. To do so, we need to show that for each $1 \le i \le m_1$ it holds that $\alpha$ satisfies $\varphi _i = \bigvee \nolimits _{j=1}^{m_{2,i}} \bigwedge \nolimits _{\ell =1}^{m_{3,i,j}} l_{i,j,\ell }$. Take an arbitrary $1 \le i \le m_1$. To derive a contradiction, assume that for each $1 \le j \le m_{2,i}$ it holds that there is some $1 \le \ell \le m_{3,i,j}$ such that $l_{i,j,\ell }$ is made false by $\alpha$. Then for each such $1 \le j \le m_{2,i}$ it holds that $|W_{C_{i,j}} \cap S| < k$. Then for each verification agent $a_i^j$, for $1 \le j \le m_{2,i}$ it holds that the top $|N|+1$ items in $L^{a}_{I \setminus S}$ (for $a = a_i^j$) form a subset of $Y'_i \cup W_X \cup F$. Then arguing as above, we get that $\pi$ would need to allocate an item of $Y'_i$ to each of the $|Y_i| = |Y'_i|+1$ agents $a_i^j$, which is a contradiction. Since *i* was arbitrary, we can conclude that $\alpha$ satisfies $\varphi$.

For the other direction, let $\alpha : X \rightarrow \{ 0,1 \}$ be a truth assignment of weight *k* that satisfies $\varphi$, and let *S* denote the set of *k* variable items $\{ w_u \mid x_u \in X, \alpha (x_u) = 1 \}$. To prove that *S* is a solution for $I_{PID }$, we define a proportional allocation $\pi$ in the instance obtained by removing *S*. First, for each selection agent $s_i$ with $1 \le i \le n-k$, we let $\pi$ allocate $f(s_i)$ and the *i*th item from $W_{X} \setminus S$ to $s_i$. Second, for each selection agent $s_{n-k+i}$ with $1 \le i \le k+1$, we let $\pi$ allocate $f(s_{n-k+i})$ and the dummy item $c_i$ to $s_{n-k+i}$. Then, for each $1 \le i \le m_1$, let $1 \le j_i \le m_{2,i}$ be some number such that $\alpha$ makes $\bigwedge \nolimits _{\ell = 1}^{m_{3,i,j_i}} l_{i,j_i,\ell }$ true—we know that such a $j_i$ exists for each *i* because $\alpha$ satisfies $\varphi$. For each verification agent $a_i^j$ we let $\pi$ allocate $f(a_i^j)$ and one item from $Y_i$ to $a_i^j$ as follows. If $j = j_i$, we let $\pi$ allocate $y_{i,m_{2,i}}$ to $a_i^j$; if $j < j_i$, we let $\pi$ allocate $y_{i,j}$ to $a_i^j$; and if $j > j_i$, we let $\pi$ allocate $y_{i,j-1}$ to $a_i^j$. It is straightforward to check that $\pi$ is indeed proportional. $\square$

Theorem 2 implies that we cannot expect an FPT-algorithm for PID with respect to the parameter *k*, the number of item deletions allowed, unless $\textsf {FPT} \ne \textsf {W[3]}$. Next we show that the brute force algorithm that runs in $|I|^{O(k)}$ time is optimal, assuming the slightly stronger assumption $\textsf {FPT} \ne \textsf {W[1]}$.

### Theorem 3

There is no algorithm for PID that on an instance $({\mathcal {P}},k)$ with item set *I* runs in $f(k) |I|^{o(k)} |{\mathcal {P}}|^{O(1)}$ time for some function *f*, unless $\mathsf {FPT} \ne {\mathsf {W}}[1]$.^2^

### Proof

Chen et al. [9] introduced the class of ${\mathsf {W}}_l[2]$-hard problems based on the notion of *linear FPT-reductions*. They proved that Dominating Set is ${\mathsf {W}}_l[2]$-hard, and that this implies a strong lower bound on its complexity: unless $\mathsf {FPT} \ne {\mathsf {W}}[1]$, Dominating Set cannot be solved in $f(k) |V|^{o(k)} (|V|+|E|)^{O(1)}$ time for any function *f*, where (*V*, *E*) is the input graph and *k* is the size of the desired dominating set.

Observe that in the FPT-reduction presented in the proof of Theorem 1, the new parameter has linear dependence on the original parameter (in fact they coincide). Therefore, this reduction is a linear FPT-reduction, and consequentially, PID is ${\mathsf {W}}_l[2]$-hard. Hence, as proved by Chen et al. [9], PID on an instance $({\mathcal {P}},k)$ with item set *I* cannot be solved in time $f(k)|I|^{o(k)} |{\mathcal {P}}|^{O(1)}$ time for any function *f*, unless $\textsf {FPT} \ne \textsf {W[1]}$. $\square$

If we want to optimize the running time not with respect to the number *k* of allowed deletions but rather in terms of the total number of items, then we can also give the following tight complexity result, under the Exponential Time Hypothesis (ETH). This hypothesis, formulated in the seminal paper by Impagliazzo, Paturi, and Zane [19] says that 3-Sat cannot be solved in $2^{o(n)}$ time, where *n* is the number of variables in the 3-CNF formula given as input.

### Theorem 4

PID can be solved in $O(2^{|I|}) \cdot |I|^{O(1)}$ time, but unless the ETH fails, it cannot be solved in $2^{o(|I|)}$ time, where *I* is the set of items in the input.

### Proof

To show that PID can be solved in $O(2^{|I|}) \cdot |I|^{O(1)}$ time, it suffices to consider the brute force algorithm that iterates over all possible subsets of items to delete, and for each such subset computes whether deleting it enables a proportional allocation (using polynomial-time matching techniques as described by Pruhs and Woeginger [23]). This algorithm runs in time $O(2^{|I|}) \cdot |I|^{O(1)}$.

Next, we show that PID cannot be solved in $2^{o(|I|)}$ time, unless the ETH fails. The so-called Sparsification Lemma proved by Impagliazzo et al. [19] implies that assuming the ETH, 3-Sat cannot be solved in $2^{o(m)}$ time, where *m* is the number of clauses in the 3-CNF formula given as input. Since the standard reduction from 3-Sat to Dominating Set transforms a 3-CNF formula with *n* variables and *m* clauses into an instance (*G*, *n*) of Dominating Set such that the graph *G* has *O*(*m*) vertices and maximum degree 3 (see, e.g., [25]), it follows that Dominating Set on a graph (*V*, *E*) cannot be solved in $2^{o(|V|)}$ time even on graphs having maximum degree 3, unless the ETH fails.

Recall that the reduction presented in the proof of Theorem 1 computes from each instance (*G*, *k*) of Dominating Set with $G=(V,E)$ an instance $({\mathcal {P}},k)$ of PID where the number of items is $3|V|+2|E|+1$. Hence, assumming that our input graph *G* has maximum degree 3, we obtain $|I|=O(|V|)$ for the set *I* of items in ${\mathcal {P}}$. Therefore, an algorithm for PID running in $2^{o(|I|)}$ time would yield an algorithm for Dominating Set running in $2^{o(|V|)}$ time on graphs of maximum degree 3, contradicting the ETH. $\square$

### Approximating PID

In view of the intractability results we have encountered sofar, it is natural to ask whether an efficient approximation might exist for PID. For some value $c \ge 1$, we say that an algorithm $\mathcal A$ is an *approximation for PID with ratio c* if, for any instance $({\mathcal {P}},k)$ of PID, ${\mathcal {A}}$ either returns a solution for ${\mathcal {P}}$ containing at most $c \cdot k$ items, or correctly concludes that there is no solution for ${\mathcal {P}}$ of size *k*.

Unfortunately, the proof of Theorem 1 implies that we cannot hope for an efficient approximation algorithm. Even if we do not aim for a constant-factor approximation, that is, for a ratio *c* for some constant *c*, but allow for a ratio $|I|^{1-\varepsilon }$ for some fixed $\varepsilon >0$, we cannot expect an efficient algorithm.

#### Theorem 5

Let $\varepsilon >0$ be a constant. If $\mathsf {FPT} \ne W[2]$, then there is no algorithm that, given an instance $({\mathcal {P}},k)$ of PID with item set *I*, yields an approximation for PID with ratio $|I|^{1-\varepsilon }$ and runs in FPT time with parameter *k*.

#### Proof

Let us suppose that ${\mathcal {A}}$ is an algorithm as described in the statement of the theorem. We are going to use ${\mathcal {A}}$ to give an FPT-algorithm for the W[2]-hard Dominating Set problem, implying $\mathsf {FPT} = W[2]$.

Let (*G*, *k*) be our instance of Dominating Set, and let *n* and *m* denote the number of vertices and edges in *G*, respectively. We first apply the reduction given in the proof of Theorem 1; let $({\mathcal {P}},k)$ be the constructed instance of PID with ${\mathcal {P}}=(N,I,L)$. Recall that $|N|=3n+2m+1$ and $|I|=2|N|+k$. We distinguish between two cases, depending on the relationship between |*N*| and *k*; recall that $\varepsilon$ is a positive constant.

First, if $|N|< 3^{\frac{1-\varepsilon }{\varepsilon }} \cdot k^{\frac{1}{\varepsilon }}$, then we apply the brute force algorithm for Dominating Set that selects *k* vertices in every possible way and checks whether they form a dominating set. By $n<|N|$, this approach takes

$$\begin{aligned} \left( {\begin{array}{c}n\\ k\end{array}}\right) O(n+m) \le \left( 3^{\frac{1-\varepsilon }{\varepsilon }} \cdot k^{\frac{1}{\varepsilon }}\right) ^k O(n+m) \end{aligned}$$

time, which is fixed-parameter tractable with parameter *k*.

Second, assume $3^{\frac{1-\varepsilon }{\varepsilon }} \cdot k^{\frac{1}{\varepsilon }} \le |N|$. In this case, we apply algorithm ${\mathcal {A}}$, which either correctly concludes that there does not exist a solution of size *k* for ${\mathcal {P}}$, or returns a solution *S* of size at most $|I|^{1-\varepsilon } k$. Observe that by $|I|=2|N|+k$ we have

$$\begin{aligned} |S|\le |I|^{1-\varepsilon } k \le (3|N|)^{1-\varepsilon } k = |N|^{1-\varepsilon } \cdot 3^{1-\varepsilon } k \le |N|^{1-\varepsilon } \cdot |N|^\varepsilon =|N| \end{aligned}$$

where the last inequality follows from our assumption on |*N*|.

Recall that ${\mathcal {P}}-S$ must contain a number of items that is a multiple of |*N*|, as otherwise no proportional allocation may exist for ${\mathcal {P}}-S$. Hence, $|S| \equiv k \mod |N|$, and thus $|S|\le |N|$ implies that *S* must be a solution of size *k*. Hence, ${\mathcal {A}}$ either finds a solution of size *k* for ${\mathcal {P}}$, or reports that no such solution exists. By the correctness of our reduction, a solution of size *k* for ${\mathcal {P}}$ implies the existence of a dominating set of size *k* for *G* (in the proof of Theorem 1 we actually determined such a set). Since ${\mathcal {A}}$ is an FPT-algorithm with parameter *k*, the presented algorithm for Dominating Set is also FPT with parameter *k*. $\square$

Inspecting the proof of Theorem 5, one can observe that the necessity of finding a solution such that the number of remaining items is a multiple of |*N*| seems to be a major impediment when considering approximation for PID. This led us to ask a different question: instead of approximating the size of the solution, is it perhaps possible to approximate the number of items that each agent ends up with in a proportional allocation? More formally, our task is the following: given a profile ${\mathcal {P}}$ and some integer *c*, determine a set *U* of items with $|U|=c |N|$ such that *U* can be proportionally allocated to the set *N* of agents (i.e., such that ${\mathcal {P}}- (I \setminus U)$ admits a proportional allocation).

Looking into the proofs of Theorems 1 and 2, we can immediately observe that the case $c=2$, that is, finding 2|*N*| items (yielding two items for each agent) for which a proportional allocation exists, is already computationally intractable.

#### Corollary 1

Given a profile ${\mathcal {P}}$ with a set *N* of agents and a set *I* of items, it is ${{\mathsf {N}}}{{\mathsf {P}}}$-hard to decide whether there exists a set of 2|*N*| items which can be proportionally allocated to the agents of *N*.

We remark that Corollary 1 directly implies that it is ${{\mathsf {N}}}{{\mathsf {P}}}$-hard to approximate the number of items each agent obtains in a proportional allocation with a ratio better than $\frac{1}{2}$.

By contrast, there is a simple algorithm to decide whether we can find one item for each agent in a proportional way.

#### Theorem 6

There exists an algorithm that given a profile ${\mathcal {P}}=(N,I,L)$ determines in polynomial time a set *U* of |*N*| items that can be proportionally allocated to the agents of *N*, whenever such a set *U* exists.

#### Proof

Suppose that *S* is a set of $|I|-|N|$ items such that ${\mathcal {P}}-S$ is solvable. Then, clearly, there cannot be two agents whose first-choice items in ${\mathcal {P}}-S$ coincide. This simple observation leads us to the following algorithm. Starting from ${\mathcal {P}}$, we repeatedly search for a pair of agents whose first-choice items coincide. If there exist such agents, then we remove their common first-choice item from ${\mathcal {P}}$ (as this item must be contained in *S*), and proceed with the remaining profile. Whenever we reach a profile such that no two agents’ first-choice items coincide, then we allocate to each agent its first-choice item (and we delete all remaining items).

Since we only delete items from *S* (except for the deletion of superfluous items performed after an appropriate allocation is found), this algorithm returns a set |*N*| of items as promised, unless ${\mathcal {P}}$ admits no solution *S* of size at most $|I|-|N|$. The running time is clearly polynomial in $|{\mathcal {P}}|$. $\square$

## Three Agents

It is known that PID for two agents is solvable in polynomial-time: if there are only two agents, then an allocation is proportional if and only it is envy-free [3]. Since the problem of obtaining an envy-free allocation by item deletion is polynomial-time solvable (in case of two agents) [4, 7], this implies tractability of PID for $|N|=2$ immediately. In this section, we generalize this result by proving that PID is polynomial-time solvable for three agents.

In what follows, we will assume that our profile ${\mathcal {P}}$ contains three agents, so let $N=\{a,b,c\}$. In Sect. 4.1, we will define the necessary basic concepts that we will need. Then, in Sect. 4.2 we present a high-level overview of our algorithm. In Section 4.3, we will look at partial solutions and define the notion of branching sets. Finally, in Sect. 4.4, when all necessary notions are in place, we present our algorithm.

### Basic Concepts: Prefixes and Minimal Obstructions

We begin by defining a graph representation of our profile ${\mathcal {P}}$ which can be used to determine whether ${\mathcal {P}}$ admits a proportional allocation. The following construction is identical to the one proposed by Pruhs and Woeginger [23, Section 4] and later generalized by Aziz et al. [3, Theorem 6].

**Fig. 1:**
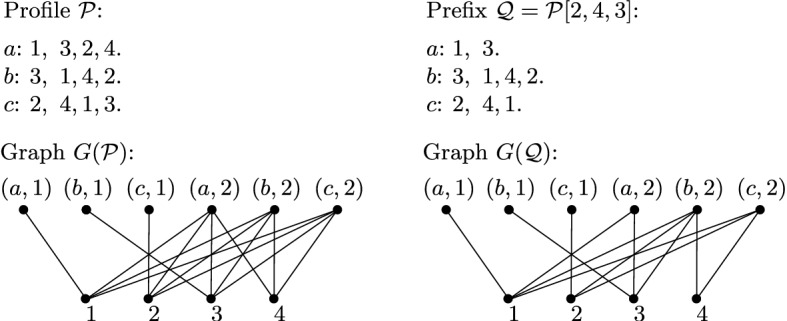
Illustration for the graph underlying a profile ${\mathcal {P}}$ and its prefix ${\mathcal {Q}}$. Note that slots (*a*, 2) and (*c*, 2) are incomplete in $G({\mathcal {Q}})$

**Graph underlying a profile** Let us define the underlying graph *G* of our profile ${\mathcal {P}}$ of PID as the following bipartite graph. The vertex set of *G* consists of the set *I* of items on the one side, and a set *S* on the other side, containing all pairs of the form (*x*, *i*) where $x \in N$ is an agent and $i \in \{1, \dots , \lceil |I|/|N| \rceil \}$. Such pairs are called *slots*. We can think of the slot (*x*, *i*) as the place for the *i*th item that agent *x* receives in some allocation. We say that an item is *eligible* for a slot (*x*, *i*), if it is contained in $L^x[1:|N|(i-1)+1]$. In the graph *G*, we connect each slot with the items that are eligible for it; see Fig. 1 for an illustration. Observe that any proportional allocation corresponds to a perfect matching in *G*; for the sake of completeness, we will prove this in Lemma 1.

Since our approach to solve PID with three agents is to apply dynamic programming, we need to handle partial instances of PID. Let us define now the basic necessary concepts.

**Prefixes** For any triple $(i_a,i_b,i_c)$ with $1 \le i_a,i_b,i_c \le |I|$ we define a *prefix* ${\mathcal {Q}}={\mathcal {P}}[i_a,i_b,i_c]$ of ${\mathcal {P}}$ as the triple $(L^a[1:i_a],L^b[1:i_b],L^c[1:i_c]$), listing only the first $i_a$, $i_b$, and $i_c$ items in the preference list of agents *a*, *b*, and *c*, respectively. We call $(i_a,i_b,i_c)$ the *size* of ${\mathcal {Q}}$ and denote it by $size ({\mathcal {Q}})$.

We say that a prefix ${\mathcal {P}}_i={\mathcal {P}}[i_a,i_b,i_c]$ is *contained in* another prefix ${\mathcal {P}}_j={\mathcal {P}}[j_a,j_b,j_c]$ if $j_x \le i_x$ for each $x \in N$; the containment is *strict* if $j_x<i_x$ for some $x \in N$. We say that ${\mathcal {P}}_i$ and ${\mathcal {P}}_j$ are *intersecting* if none of them contains the other; we call the unique largest prefix contained both in ${\mathcal {P}}_i$ and in ${\mathcal {P}}_j$, i.e., the prefix ${\mathcal {P}}[\min (i_a,j_a),\min (i_b,j_b),\min (i_c,j_c)]$, their *intersection*, and denote it by ${\mathcal {P}}_i \cap {\mathcal {P}}_j$. We may also compare prefixes of different profiles, deciding their relationship (i.e., whether one contains the other, or they intersect) solely based on their sizes.

For some prefix ${\mathcal {Q}}={\mathcal {P}}[i_a,i_b,i_c]$, let $I({\mathcal {Q}})$ denote the set of all items appearing in ${\mathcal {Q}}$. We define the set of slots appearing in ${\mathcal {Q}}$ as $S({\mathcal {Q}})=\{ (x,i) \mid 1 \le i \le \lceil (i_x+2)/3 \rceil , x \in N\}$. We also define the graph $G({\mathcal {Q}})$ underlying ${\mathcal {Q}}$ as the subgraph of *G* where a slot $(x,i) \in S({\mathcal {Q}})$ is adjacent to an item $u \in I({\mathcal {Q}})$ if *u* appears in $L^x[1:i_x]$ and is eligible for (*x*, *i*) in *G*; see Fig. 1 for an illustration. Note that any slot (*x*, *i*) where $1 \le i \le \lfloor (i_x+2)/3 \rfloor$ is connected to the same items in $G({\mathcal {Q}})$ as in *G*; we say that such slots are *complete* in ${\mathcal {Q}}$. By contrast, if $i_x \not \equiv 1 \mod 3$, then the slot $(x, \lceil (i_x+2)/3 \rceil )$ is connected to fewer items in $G({\mathcal {Q}})$ than in *G*. Hence, the only slots which may be incomplete are the last slots in ${\mathcal {Q}}$, that is, the slots $(x,\lceil (i_x+2)/3 \rceil )$, $x \in N$. See Fig. 1 for an illustration.

**Solvability** We say that a prefix ${\mathcal {Q}}$ is *solvable*, if the underlying graph $G({\mathcal {Q}})$ has a matching that covers all its complete slots. Hence, a prefix is solvable exactly if there exists an allocation $\pi$ from $I({\mathcal {Q}})$ to *N* that satisfies the condition of proportionality restricted to each index in ${\mathcal {Q}}$: for any agent $x \in N$ and any index $i \in \{1, \dots , i_x \}$, the number of items from $L^x[1:i]$ allocated by $\pi$ to *x* is at least *i*/3.

**Fig. 2:**
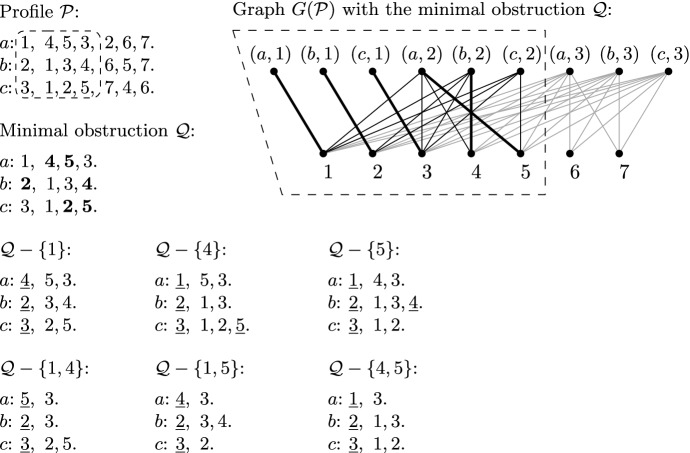
Illustration depicting a profile ${\mathcal {P}}$ with the graph $G({\mathcal {P}})$ (edges incident to the last slots are grey only to help visibility). The matching in $G({\mathcal {P}})$ shown in bold is the one found by the algorithm of Lemma 1; observe that there is no augmenting path from (*c*, 2). The slots reachable in $G({\mathcal {P}})$ by alternating paths from slot (*c*, 2) together with all items eligible for them (as depicted in $G({\mathcal {P}})$ by the dashed trapezoid) yield the minimal obstruction ${\mathcal {Q}}$ of straight shape. The boundary of ${\mathcal {Q}}$ is $\delta ({\mathcal {Q}})=\{2,4,5\}$ (emphasized in bold). There are six partial solutions for ${\mathcal {Q}}$ of size at most 2, namely $\{1\}$, $\{4\}$, $\{5\}$, $\{1,4\}$, $\{1,5\}$, and $\{4, 5\}$. Each partial solution *U* is witnessed by an allocation $\pi _U$ showing that ${\mathcal {Q}}-U$ is solvable; in each (partial) preference list for ${\mathcal {Q}}-U$, we indicated the items allocated by $\pi _U$ to the given agent by underlining them

**Minimal obstructions** We say that a prefix ${\mathcal {Q}}$ is a *minimal obstruction*, if it is not solvable, but all prefixes strictly contained in ${\mathcal {Q}}$ are solvable. Observe that all slots in a minimal obstruction must be complete. Furthermore, Hall’s Theorem tells us that a minimal obstruction must have exactly one item less than the number of slots, so $|I({\mathcal {Q}})| = |S({\mathcal {Q}})|-1$. We will call any prefix ${\mathcal {Q}}$ that is not solvable an *obstruction*; note that any obstruction that does not strictly contain another obstruction is, indeed, a minimal obstruction in the above sense. See Fig. 2 for an illustration. Lemma 1 shows that a minimal obstruction, if existing, can be found efficiently; Lemma 2 states some useful observations about minimal obstructions.

#### Lemma 1

Profile ${\mathcal {P}}$ admits a proportional allocation if and only if the underlying graph *G* contains a perfect matching. Also, in $O(|I|^3)$ time we can find either a proportional allocation for ${\mathcal {P}}$, or a minimal obstruction ${\mathcal {Q}}$ in ${\mathcal {P}}$.

#### Proof

We prove this lemma for arbitrary |*N*|.

First, it is easy to see that any proportional allocation $\pi$ immediately yields a perfect matching *M* for *G*: for each $x \in N$ and each $i \in \{1, \dots , |I|/|N|\}$ (note that $|I|/|N| \in {\mathbb {N}}$ since $\pi$ is proportional), we simply put into *M* the edge connecting slot (*x*, *i*) with the *i*th item $p_{(x,i)}$ received by *x*; naturally, we rank items received by *x* according to *x*’s preferences. The proportionality of $\pi$ implies that $p_{(x,i)}$ is contained in the top $(i-1)|N|+1$ items in $L^x$, and thus is indeed eligible for the slot (*x*, *i*).

For the other direction, consider a perfect matching *M* in *G*. Then giving each agent *x* all the items assigned to the slots $\{(x,i) \mid i \in \{1, \dots , |I|/|N|\}$ by *M* we obtain a proportional allocation $\pi$: for each agent *x* and index $j \in \{1, \dots , |N|\}$, our allocation $\pi$ assigns at least *j*/|*N*| items to *x* from $L^x[1:j]$, namely the items matched by *M* to the slots $\{(x,i) \mid 1 \le i \le \lceil j/|N| \rceil \}$. Since $(\lceil j/|N| \rceil -1)|N|+1 \le j$, even the last item eligible for $(x, \lceil j/|N| \rceil )$ is contained in $L^x[1:j]$, ensuring that $\pi$ is indeed proportional.

Therefore, we can check whether there exists a proportional allocation for ${\mathcal {P}}$ by finding a maximum matching in the bipartite graph *G*. Using the Hopcroft–Karp algorithm [17], this takes $O(|I|^{5/2})$ time, since *G* has 2|*I*| vertices. If no perfect matching exists in *G*, then we can find a minimal obstruction using a variant of the classical augmenting path method that starts from an empty matching, and increases its size by finding augmenting paths one by one. Namely, at each iteration we pick an unmatched starting slot (*x*, *i*) for which all slots in $\{(x',j)\mid x' \in N, 1 \le j < i \}$ are already matched, and search for an augmenting path that starts at (*x*, *i*).

Suppose that this algorithm stops at an iteration where the starting slot is (*x*, *i*), and no augmenting path starts at (*x*, *i*) for the current matching *M*. Let $S_H$ be the set of all slots reachable by an alternating path in *G* from (*x*, *i*), and let $I_H$ be the set of all items eligible for any slot in $S_H$. It is well known that $S_H$ and $I_H$ violate Hall’s condition: $|I_H|<|S_H|$. Moreover, the slots in $S_H$ “induce” a prefix in the sense that there exists a prefix ${\mathcal {Q}}$ with $S({\mathcal {Q}})=S_H$. To prove this, it suffices to show that if $(y,j) \in S_H$ and $j' \in \{1, \dots , j-1\}$, then $(y,j') \in S_H$. By our strategy for picking starting slots, we know $j' <j \le i$, implying that $(y,j')$ is matched by *M*. Let *q* be the item assigned to it by *M*; note that *q* is eligible for (*y*, *j*) as well. To obtain an alternating path from (*x*, *i*) to $(y,j')$, we can take any alternating path from (*x*, *i*) to (*y*, *j*), and append the two-edge path from (*y*, *j*) to $(y,j')$ through *q*. Hence, there indeed exists a prefix ${\mathcal {Q}}$ with $S({\mathcal {Q}})=S_H$; we pick such a ${\mathcal {Q}}$ containing only complete slots. Using standard arguments from matching theory, it is straightforward to check that ${\mathcal {Q}}$ is a minimal obstruction.

Each iteration can be performed in $O(|I|^2)$ time (e.g., with a BFS), and there are at most |*I*| steps, so the algorithm runs in $O(|I|^3)$ time. $\square$

#### Lemma 2

Let ${\mathcal {Q}}={\mathcal {P}}[i_a,i_b,i_c]$ be a prefix of ${\mathcal {P}}$ that is a minimal obstruction. Then $i_a \equiv i_b \equiv i_c \equiv 1 \mod 3$, $|I({\mathcal {Q}})|=(i_a+i_b+i_c)/3+1$, and either

(i) $$i_a=i_b=i_c$$, or
(ii) $i_x=i_y=i_z+3$ for some choice of agents *x*, *y*, and *z* with $\{x,y,z\}=\{a,b,c\}$.

Moreover, if (ii) holds, then $L^x[1:i_x]$ and $L^y[1:i_y]$ contain exactly the same item set, namely $I({\mathcal {Q}})$.

#### Proof

First, observe that if $i_a \not \equiv 1 \mod 3$, then the set of complete slots is the same in ${\mathcal {Q}}$ as in ${\mathcal {P}}[i_a-1,i_b,i_c]$, contradicting the minimality of ${\mathcal {Q}}$. Thus, we have $i_a \equiv 1 \mod 3$, and we get $i_b \equiv i_c \equiv 1 \mod 3$ analogously.

Second, let us consider the graph $G({\mathcal {Q}})$ underlying our prefix. Since Hall’s condition fails for the set $S({\mathcal {Q}})$ of (complete) slots but, by minimality, it holds for any proper subset of these slots, we know that

1 $$\begin{aligned} |I({\mathcal {Q}})|=|S({\mathcal {Q}})|-1= \left\lceil \frac{i_a+2}{3} \right\rceil + \left\lceil \frac{i_b+2}{3} \right\rceil + \left\lceil \frac{i_c+2}{3} \right\rceil -1 = \frac{i_a+i_b+i_c}{3}+1 \end{aligned}$$

where the last equality follows from the first claim of the lemma. Let us assume $i_a \ge \max \{ i_b,i_c\}$. Note that if neither (i) nor (ii) holds, then by the maximality of $i_a$ and the first claim of the lemma we obtain $i_a+i_b+i_c \le 3i_a -6$, from which (1) implies $|I({\mathcal {Q}})| \le i_a-1$. However, $L^a[1:i_a]$ contains only items from $I({\mathcal {Q}})$, which would imply that some item appears twice in $L^a[1:i_a]$, a contradiction.

To see the last claim of the lemma, suppose $i_a=i_b=i_c+3$. Then (1) implies $|I({\mathcal {Q}})|=i_a=i_b$, and hence $L^a[1:i_a]$ (and also $L^b[1:i_b]$) must contain each item in $I({\mathcal {Q}})$ exactly once. $\square$

**Fig. 3:**
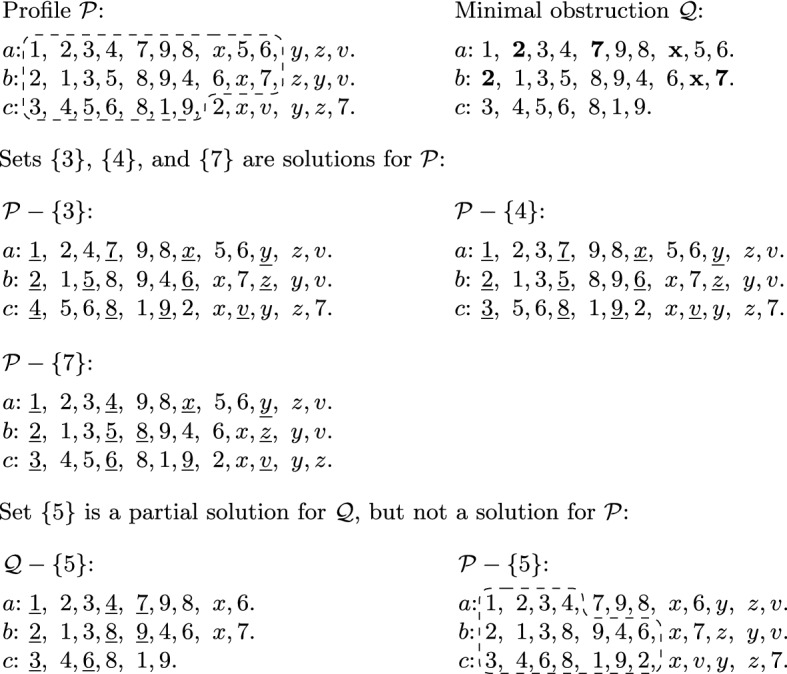
Illustration depicting a profile ${\mathcal {P}}$ containing a minimal obstruction ${\mathcal {Q}}$ of slant shape. Proportional allocations, where existent, are indicated by underlining. We investigate four partial solutions, $\{3\}$, $\{4\}$, $\{5\}$ and $\{7\}$, for ${\mathcal {Q}}$. The boundary of ${\mathcal {Q}}$ is $\delta ({\mathcal {Q}})=\{2,7,x\}$ (emphasized in bold). Deleting either 3, 4, or 7 yields a solution for ${\mathcal {P}}$, but deleting item 5 does not; we have depicted a minimal obstruction in ${\mathcal {P}}-\{5\}$

Based on Lemma 2, we define the *shape* of a minimal obstruction ${\mathcal {Q}}$ as either *straight* or *slant*, depending on whether ${\mathcal {Q}}$ fulfills the conditions (i) or (ii), respectively. More generally, we also say that a prefix has straight or slant shape if it fulfills the respective condition. Furthermore, we define the *boundary items* of ${\mathcal {Q}}$, denoted by $\delta ({\mathcal {Q}})$, as the set of all items that appear once or twice (but not three times) in ${\mathcal {Q}}$. Fig. 2 depicts a minimal obstruction of straight shape, while Fig. 3 shows one of slant shape; both examples indicate the boundary of the minimal obstruction as well.

#### Lemma 3

Let ${\mathcal {Q}}$ be a prefix of ${\mathcal {P}}$ that is a minimal obstruction. Then the boundary of ${\mathcal {Q}}$ contains at most three items: $|\delta ({\mathcal {Q}})| \le 3$.

#### Proof

We make use of Lemma 2. First, if ${\mathcal {Q}}$ has a straight shape, so ${\mathcal {Q}}={\mathcal {P}}[i,i,i]$ for some index *i*, then $|S({\mathcal {Q}})|=i+2$. Since ${\mathcal {Q}}$ is a minimal obstruction, by Lemma 2 we get $|I({\mathcal {Q}})| = i+1$. However, each agent’s list within ${\mathcal {Q}}$ contains exactly *i* items, yielding that there is exactly one position outside ${\mathcal {Q}}$ in each agent’s list where an item of $I({\mathcal {Q}})$ occurs. Hence, $|\delta ({\mathcal {Q}})| \le 3$ follows in this case.

Second, assume that ${\mathcal {Q}}$ has a slant shape, say ${\mathcal {Q}}={\mathcal {P}}[i,i,i-3]$ for some index *i* (the two remaining cases are analogous). Then Lemma 2 implies $|I({\mathcal {Q}})|=i$ and that both $L^a[1:i]$ and $L^b[1:i]$ contain all the *i* items in $I({\mathcal {Q}})$, but $L^c[1:i-3]$ misses exactly three items from $I({\mathcal {Q}})$. Hence, there are exactly three occurrences of items listed outside ${\mathcal {Q}}$, each in the list of agent *c*, meaning $|\delta ({\mathcal {Q}})|=3$. $\square$

### High-level Overview of Our Algorithm

Having in place the most basic definitions, we are now able to give an intuition about how our algorithm works. The main idea is to repeatedly find a minimal obstruction, delete certain items from it to render it solvable (i.e., to ensure that the remainder admits a proportional allocation), and then proceed with the modified instance.

However, we are not able to immediately tell which items should be deleted from the current minimal obstruction ${\mathcal {Q}}$, as such a decision may have consequences later, when we are dealing with subsequent minimal obstructions. Therefore, instead of picking just one solution, we apply a *bounded search tree* approach: at each minimal obstruction, we perform a branching, and pursue several possible ways to delete a set of items to make ${\mathcal {Q}}$ solvable. To obtain a polynomial-time algorithm, we must bound the size of our search tree; for this we need several ideas.

**Bounding the number of branches** In order to bound the number of branches that we have to investigate in a branching, we use the important fact stated by Lemma 4 that *any minimal solution removes at most two items* from a minimal obstruction. This insight is of crucial importance in our algorithm, as it yields a polynomial bound on the number of branches, namely $O(|I|^2)$.

**Bounding the size of the search tree** Although our search tree algorithm has a recursive structure, we apply a *dynamic programming technique* to limit the number of recursive calls, i.e., the number of nodes in the search tree.

To this end, we define an *equivalence relation between partial solutions*, corresponding to nodes in the search tree. Intuitively, two partial solutions are equivalent if they can be extended in the same way into a solution. It turns out that we can determine sufficient conditions that guarantee equivalence. These conditions are somewhat technical, but they essentially ensure that two deletions have the same effect with respect to any possible minimal obstruction that may arise later during the run of the algorithm.

These conditions allow us to classify partial solutions into equivalence classes whose number is bounded by a polynomial; this results in a polynomial running time for our algorithm.

### Partial Solutions and Branching Sets

**Partial solutions** For a prefix ${\mathcal {Q}}$ and a set *U* of items, we define ${\mathcal {Q}}-U$ in the natural way: by deleting all items of *U* from the (partial) preference lists of the prefix (note that the total length of the preference lists constituting the prefix may decrease). We say that an item set $Y \subseteq I({\mathcal {Q}})$ is a *partial solution for* ${\mathcal {Q}}$ if ${\mathcal {Q}}-Y$ is solvable. See again Fig. 2 or 3 for an example. Observe that for any item set *Y* we can check whether it is a partial solution for ${\mathcal {Q}}$ by checking whether all complete slots can be covered by a matching in the graph corresponding to ${\mathcal {Q}}-Y$.

**Branching set.** To solve PID we will repeatedly apply a branching step: whenever we encounter a minimal obstruction ${\mathcal {Q}}$, we shall consider several possible partial solutions for ${\mathcal {Q}}$, and for each partial solution *Y* we try to find a solution *U* for ${\mathcal {P}}$ such that $U\cap I({\mathcal {Q}}) =Y$. To formalize this idea, we say that a family ${\mathcal {Y}}$ containing partial solutions for a minimal obstruction ${\mathcal {Q}}$ is a *branching set for* ${\mathcal {Q}}$, if there exists a solution *U* of minimum size for the profile ${\mathcal {P}}$ such that $U \cap I({\mathcal {Q}}) \in {\mathcal {Y}}$. Such a set is exactly what we need to build a search tree algorithm for PID.

Lemma 4 shows that we never need to delete more than two items from any minimal obstruction. This will be essential for constructing a branching set.

#### Lemma 4

Let ${\mathcal {Q}}$ be a minimal obstruction in a profile ${\mathcal {P}}$, and let *U* denote an inclusion-wise minimal solution for ${\mathcal {P}}$. Then $|U \cap I({\mathcal {Q}})| \le 2$.

#### Proof

Let $U_{{\mathcal {Q}}}:=U \cap I({\mathcal {Q}})$, and let us assume $|U_{{\mathcal {Q}}}| \ge 3$ for contradiction. We are going to select a set *Y* of three items from $U_{{\mathcal {Q}}}$ for which we can prove that $U \setminus Y$ is a solution for ${\mathcal {P}}$, contradicting the minimality of *U*.

We rank the items of $U_{{\mathcal {Q}}}$ according to the index of the first slot in which they appear in ${\mathcal {P}}$: we say that an item *u* *appears at* *i*, if *i* is the smallest index such that *u* is eligible for a slot (*x*, *i*) for some $x \in N$. If there exist three items $y_1$, $y_2$, and $y_3$ in $U_{{\mathcal {Q}}}$ appearing strictly earlier (i.e., at a smaller index) than all other items in $U_{{\mathcal {Q}}}$, then we let $Y=\{y_1,y_2,y_3\}$.

Otherwise, we apply the following procedure to choose *Y*. Let $Y_1$ be the set of items in $U_{{\mathcal {Q}}}$ that appear at the earliest index, say $i_1$. We select $y_1$ from $Y_1$ by favoring items eligible for more than one slot from $\{(a,i_1),(b,i_1),(c,i_1)\}$; if there are still several possibilities to choose $y_1$, then we select it arbitrarily. Similarly, let $Y_2$ be the set of earliest appearing items in $U_{{\mathcal {Q}}} \setminus \{y_1\}$, appearing at some index $i_2$. We pick an item $y_2$ from $Y_2$ by favoring items eligible for more than one slot from $\{(a,i_2),(b,i_2),(c,i_2)\}$; again, if there are still several possibilities to choose $y_2$, then we select it arbitrarily. Note that we use the notion of eligibility based on the original preference lists in ${\mathcal {P}}$.

To choose an item $y_3$ from the set $Y_3$ of the earliest appearing items in $U_{{\mathcal {Q}}} \setminus \{y_1,y_2\}$, we create the profile ${\mathcal {P}}_3={\mathcal {P}}-(U \setminus \{y_1,y_2\})$. If there exists a minimal obstruction in ${\mathcal {P}}_3$ strictly contained in ${\mathcal {Q}}$, then we fix such a minimal obstruction ${\mathcal {Q}}_3$, and we choose an item $y_3 \in Y_3$ eligible for a slot of $S({\mathcal {Q}}_3)$. Otherwise we choose $y_3$ from $Y_3$ arbitrarily. Intuitively, we choose $y_3$ so as to overcome the possible obstructions obtained when putting $y_1$ and $y_2$ back into our instance, and our strategy for this is simply to choose an item lying within any such obstruction. Observe that if the minimal obstruction ${\mathcal {Q}}_3$ exists, then (1) since there is no obstruction strictly contained in ${\mathcal {Q}}$ in the profile ${\mathcal {P}}-U_{{\mathcal {Q}}}$, there must exist some item in $U_{{\mathcal {Q}}} \setminus \{y_1,y_2\}$ that is eligible for some slot in $S({\mathcal {Q}}_3)$; and (2) if *u* appears earliest in ${\mathcal {P}}$ among all such items, then $u \in Y_3$. To see this, let (*x*, *i*) be the first slot in $S({\mathcal {Q}}_3)$ for which *u* is eligible in ${\mathcal {P}}_3$. By the claim of Lemma 2 on the shape of a minimal obstruction, all slots preceding (*x*, *i*) belong to ${\mathcal {Q}}_3$ as well, that is, the prefix ${\mathcal {P}}_3^{<i}={\mathcal {P}}_3[3i-5,3i-5,3i-5]$ “induced” by these slots in ${\mathcal {P}}_3$ is contained in ${\mathcal {Q}}_3$. Thus, by our choice of *u*, we get that ${\mathcal {P}}_3^{<i}$ is a prefix of ${\mathcal {P}}$ as well, implying that no item of $U_{{\mathcal {Q}}} \setminus \{y_1,y_2\}$ appears earlier in ${\mathcal {P}}$ than *u*. Hence, $u \in Y_3$, showing that $y_3$ is well-defined.

Setting $Y=\{y_1,y_2,y_3\}$, we finish our proof by proving that $U \setminus Y$ is a solution for ${\mathcal {P}}$. For contradiction, suppose that ${\mathcal {R}}$ is a minimal obstruction in ${\mathcal {P}}'={\mathcal {P}}-(U \setminus Y)$.

First, suppose that ${\mathcal {R}}$ contains all items in *Y*. As *U* is a solution, the profile ${\mathcal {R}}-Y$ is solvable, and hence contains at least as many items as complete slots. Note that adding the items of *Y* into the profile ${\mathcal {R}}-Y$ means adding exactly three new items and at most three new complete slots (since each agent’s list contains at most three more items, resulting in at most one extra complete slot per agent). Hence, ${\mathcal {R}}$ has at least as many items as slots, contradicting the assumption that ${\mathcal {R}}$ is a minimal obstruction.

Hence we know that ${\mathcal {R}}$ does not contain all items in *Y*. By Lemma 2, ${\mathcal {R}}$ is then strictly contained^3^ in ${\mathcal {Q}}$, and by the minimality of ${\mathcal {Q}}$ we get that ${\mathcal {R}}$ must contain an item from $\{y_1,y_2,y_3\}$. We claim that if ${\mathcal {R}}$ contains $y_h$ for some $h \in \{2,3\}$, then it contains all items $y_j$ with $1 \le j < h$. Since $y_j$ appears not later than $y_h$, the only possible way for ${\mathcal {R}}$ to contain $y_h$ but *not* $y_j$ would be the following: $y_j$ and $y_h$ appear at the same slot number *i*, but ${\mathcal {R}}$ has a slant shape and thus only contains two slots from $S_i:=\{ (x,i) \mid x \in N\}$, missing exactly the (unique) slot where $y_j$ appears. However, since ${\mathcal {R}}$ is a minimal obstruction, $y_h$ must appear at *both* remaining slots from $S_i$ by the last statement of Lemma 2, which contradicts our choice of $y_j$.

This leaves us with the case when $y_3$ is not contained in ${\mathcal {R}}$ (for $y_3 \in I({\mathcal {R}})$ would imply $Y \subseteq I({\mathcal {R}})$, which we already proved not to be the case). Then ${\mathcal {R}}$ is not only a prefix of ${\mathcal {P}}'$ but also of ${\mathcal {P}}_3$. Assume w.l.o.g. that $y_3$ appears at index *j* in the slot (*c*, *j*). Since ${\mathcal {R}}$ is a minimal obstruction in ${\mathcal {P}}_3$ strictly contained in ${\mathcal {Q}}$, we know that a minimal obstruction ${\mathcal {Q}}_3$ was found when choosing $y_3$, but ${\mathcal {R}} \ne {\mathcal {Q}}_3$. Thus, both ${\mathcal {R}}$ and ${\mathcal {Q}}_3$ are minimal obstructions of slant shape, with ${\mathcal {R}}$ containing the slots (*a*, *j*) and (*b*, *j*) but not (*c*, *j*), and ${\mathcal {Q}}_3$ containing the slot (*c*, *j*) and one of (*a*, *j*) and (*b*, *j*), say (*b*, *j*). This means that ${\mathcal {R}}={\mathcal {P}}_3[3j-2,3j-2,3j-5]$ and ${\mathcal {Q}}_3={\mathcal {P}}_3[3j-5,3j-2,3j-2]$. Note also that by the last statement of Lemma 2, we know

$$\begin{aligned} L^a_{I \setminus (U\setminus \{y_1,y_2\})}[1:3j-2]=L^b_{I \setminus (U\setminus \{y_1,y_2\})}[1:3j-2]=L^c_{I \setminus (U\setminus \{y_1,y_2\})}[1:3j-2]. \end{aligned}$$

This means that $P_3[3j-2,3j-2,3j-2]$ contains exactly $3j-2$ items.

Observe that deleting $\{y_1,y_2\}$ from profile ${\mathcal {P}}_3[3j-2,3j-2,3j-2]$ results in a prefix ${\mathcal {T}}$ of ${\mathcal {P}}_3-\{y_1,y_2\}={\mathcal {P}}-U$ of size $[3j-4,3j-4,3j-4]$ that contains exactly $3j-4$ items. However, $S({\mathcal {T}})$ contains $3j-3$ complete slots (and 3 incomplete ones). Therefore, ${\mathcal {P}}-U$ contains a prefix that is not solvable, a contradiction finishing the proof. $\square$

Lemma 4 implies that simply taking all partial solutions of $I({\mathcal {Q}})$ of size 1 or 2 yields a branching set for ${\mathcal {Q}}$. As an example, the minimal obstruction shown in Fig. 2 admits the branching set $\{ \{1\},\{4\},\{5\},\{1,4\},\{1,5\},\{4,5\} \}$.

#### Corollary 2

For any minimal obstruction ${\mathcal {Q}}$ in a profile, a branching set ${\mathcal {Y}}$ for ${\mathcal {Q}}$ of cardinality at most $$|I({\mathcal {Q}})|+\left( {\begin{array}{c}|I({\mathcal {Q}})|\\ 2\end{array}}\right) =O(|I|^2)$$ and with $\max _{Y \in {\mathcal {Y}}} |Y| \le 2$ can be constructed in $O(|I|^4)$ time.

#### Proof

By Lemma 4, in order to construct the branching set ${\mathcal {Y}}$ as required, it suffices to check for each $Y \subseteq I({\mathcal {Q}})$ of size at most 2 whether ${\mathcal {Q}}-Y$ is solvable. To do so, we first construct the graph *G* underlying the prefix ${\mathcal {Q}}$ and compute a maximum matching *M* in *G*. This can be done in $O(|I|^{5/2})$ time using the Hopcroft–Karp algorithm, as explained in Lemma 1. Note that since ${\mathcal {Q}}$ is a minimal obstruction, it matches all but one slots in *G*, so $|M|=|S({\mathcal {Q}})|-1$.

Now, for each $Y \subseteq I({\mathcal {Q}})$ with $1 \le |Y| \le 2$ we compute the graph $G_Y$ underlying the prefix ${\mathcal {Q}}-Y$. Observe that we can obtain $G_Y$ from *G* by deleting the items of *Y*, and adding the necessary edges so that every slot is connected with all items eligible for it. Observe that *M* yields a matching $M_Y$ of size at least $|M|-2$ in $G_Y$, which covers at least $|M|-5=|S({\mathcal {Q}})|-6$ complete slots (because at most three slots may have become incomplete in ${\mathcal {Q}}-Y$). Hence, starting from $M_Y$ we only need to find a constant number of augmenting paths in order to check whether all complete slots of $G_Y$ can be covered by a matching. This takes $O(|I|^2)$ time, because $G_Y$ has at most 2|*I*| vertices, yielding a running time of $O(|I|^4)$ in total. $\square$

### Polynomial-Time Algorithm for PID for Three Agents

Let us now present our algorithm for solving PID on our profile ${\mathcal {P}}=(N,I,L)$.

We are going to build the desired solution step-by-step, iteratively extending an already found partial solution. For a prefix ${\mathcal {T}}$ of ${\mathcal {P}}$ and a partial solution *U* for ${\mathcal {T}}$, we call a set $E \subseteq I$ an *extension* for $({\mathcal {T}},U)$ if *E* is disjoint from $I({\mathcal {T}})$ and $E \cup U$ is a solution for ${\mathcal {P}}$; we will refer to the set of items in $I({\mathcal {T}}) \setminus U$ as *forbidden* w.r.t. $({\mathcal {T}},U)$. We propose an algorithm $Extend ({\mathcal {T}},U)$ that, given a prefix ${\mathcal {T}}$ of ${\mathcal {P}}$ and a partial solution *U* for ${\mathcal {T}}$, returns an extension for $({\mathcal {T}},U)$ of minimum size if one exists, otherwise returns ‘No’.

**Fig. 4:**
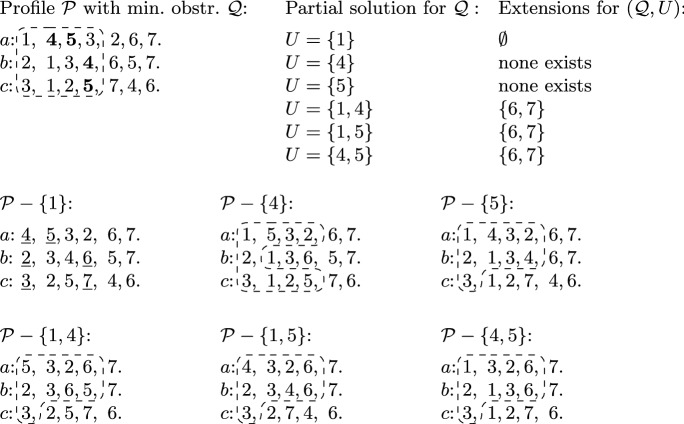
An example showing how different partial solutions for minimal obstruction ${\mathcal {Q}}$ can be extended into a solution for profile ${\mathcal {P}}$. Among all partial solutions, only $\{1\}$ is a solution for ${\mathcal {P}}$, deleting any other partial solution leads to a new minimal obstruction. Note that both 4 and 5 are contained in the boundary, while 1 is not; hence, the size of ${\mathcal {Q}}-\{1\}$ is different from that of ${\mathcal {Q}}-\{4\}$ or ${\mathcal {Q}}-\{5\}$ (as can be seen on Fig. 2). This implies that the prefixes $({\mathcal {P}}-\{4\})[4,1,4]$ and $({\mathcal {P}}-\{5\})[4,4,1]$ have less items than the corresponding prefixes in ${\mathcal {P}}-\{1\}$ of the same size, ultimately leading to the fact that $\{1\}$ is a solution for ${\mathcal {P}}$, while neither $\{4\}$ nor $\{5\}$ can be extended into a solution for ${\mathcal {P}}$ (because there exists no partial solution for the minimal obstructions depicted in ${\mathcal {P}}-\{4\}$ and ${\mathcal {P}}-\{5\}$ that is disjoint from $I({\mathcal {Q}})$). The list of possible extensions shows that $\{4\}$ is equivalent with $\{5\}$, and all partial solutions of size 2 are equivalent with each other. (We remark that, however, all partial solutions for ${\mathcal {Q}}$ have distinct deficiency patterns, so no two of them are strongly equivalent)

**Branching set with forbidden items.** To address the problem of finding an extension for $({\mathcal {T}},U)$, we modify the notion of a branching set accordingly. Given a minimal obstruction ${\mathcal {Q}}$ in some profile ${\mathcal {P}}'$ and a set $F \subseteq I({\mathcal {Q}})$ of items, we say that a family ${\mathcal {Y}}$ of partial solutions for ${\mathcal {Q}}$ is a *branching set for* ${\mathcal {Q}}$ *forbidding* *F*, if the following holds: either there exists a solution *U* for the profile ${\mathcal {P}}'$ that is disjoint from *F* and has minimum size among all such solutions, and moreover, fulfills $U \cap I(Q) \in {\mathcal {Y}}$, or ${\mathcal {P}}$ does not admit any solution disjoint from *F* (in which case ${\mathcal {Y}}$ can be arbitrary).

#### Lemma 5

There is an algorithm that, given a minimal obstruction ${\mathcal {Q}}$ in a profile and a set $F \subseteq I({\mathcal {Q}})$ of forbidden items, produces a branching set ${\mathcal {Y}}$ forbidding *F* with $\max _{Y \in {\mathcal {Y}}} |Y| \le 2$ and $|{\mathcal {Y}}|=O(|I|^2)$, and runs in time $O(|I|^4)$.

#### Proof

The algorithm given in Corollary 2 can be adapted in a straightforward fashion to take forbidden items into account: it suffices to simply discard in the first place any subset $Y \subseteq I({\mathcal {Q}})$ that is not disjoint from *F*. It is easy to verify that this modification indeed yields an algorithm as desired. $\square$

**Equivalent partial solutions.** We will describe $$Extend $$ as a recursive algorithm, but in order to ensure that it runs in polynomial time, we need to apply dynamic programming. For this, we need a notion of equivalence: we say that two partial solutions $$U_1$$ and $$U_2$$ for $${\mathcal {T}}$$ are *equivalent* if

1. $$|U_1|=|U_2|$$, and
2. $({\mathcal {T}},U_1)$ and $({\mathcal {T}},U_2)$ admit the same extensions.

See Fig. 4 for an illustration.

Ideally, whenever we perform a call to $$Extend $$ with a given input $$({\mathcal {T}},U)$$, we would like to first check whether an equivalent call has already been performed, i.e., whether $$Extend $$ has been called with an input $$({\mathcal {T}},U')$$ for which *U* and $$U'$$ are equivalent. However, the above definition of equivalence is computationally hard to handle: there is no easy way to check whether two partial solutions admit the same extensions or not. To overcome this difficulty, we will use a stronger condition that implies equivalence.

**Fig. 5:**
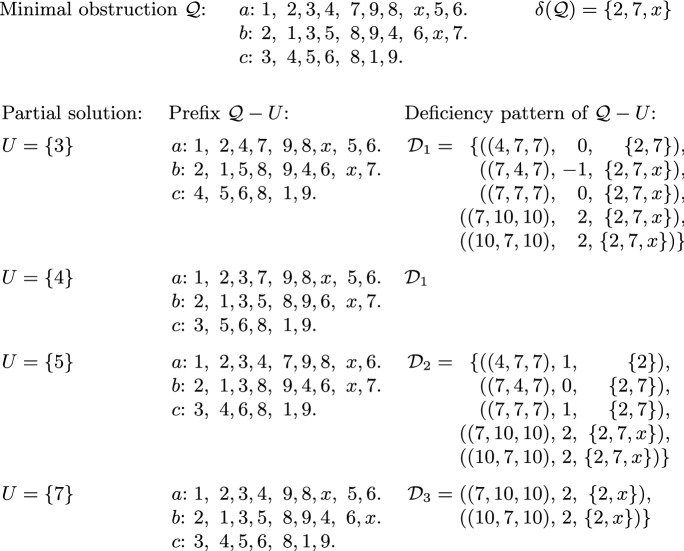
Illustration of deficiency patterns. For the minimal obstruction ${\mathcal {Q}}$ contained in profile ${\mathcal {P}}$ from Fig. 3, we show four partial solutions and the corresponding deficiency patterns. Since ${\mathcal {Q}}-\{3\}$ and ${\mathcal {Q}}-\{4\}$ have the same deficiency pattern ${\mathcal {D}}_1$, and neither 3, nor 4 is contained in the boundary $\delta ({\mathcal {Q}})$, we get that $\{3\}$ and $\{4\}$ are strongly equivalent partial solutions for ${\mathcal {Q}}$; by Lemma 6 they are also equivalent. By ${\mathcal {D}}_1 \ne {\mathcal {D}}_2$ sets $\{3\}$ and $\{5\}$ are not strongly equivalent for ${\mathcal {Q}}$. Since $7 \in \delta ({\mathcal {Q}})$, set $\{7\}$ is not strongly equivalent with any other partial solution; nevertheless, $\{3\}$ and $\{7\}$ are in fact equivalent with respect to ${\mathcal {Q}}$: they are both solutions for ${\mathcal {P}}$ and thus admit the extension $\emptyset$ (recall Fig. 3)

**Deficiency patterns** Consider a solvable prefix ${\mathcal {Q}}$ of ${\mathcal {P}}$. We let the *deficiency* of ${\mathcal {Q}}$, denoted by $def ({\mathcal {Q}})$, be the value $|S({\mathcal {Q}})|-|I({\mathcal {Q}})|$. Note that due to possibly incomplete slots in ${\mathcal {Q}}$, the deficiency of ${\mathcal {Q}}$ may be positive even if ${\mathcal {Q}}$ is solvable. However, if ${\mathcal {Q}}$ contains only complete slots, then its solvability implies $def ({\mathcal {Q}}) \le 0$. We define the *deficiency pattern* of ${\mathcal {Q}}$, denoted by $defpat (Q)$, as the set of all triples

$$\begin{aligned} (size ({\mathcal {R}}),def ({\mathcal {Q}}\cap {\mathcal {R}}),I({\mathcal {Q}}\cap {\mathcal {R}}) \cap \delta (Q)) \end{aligned}$$

where ${\mathcal {R}}$ can be any prefix with a straight or a slant shape that intersects ${\mathcal {Q}}$. Roughly speaking, the deficiency pattern captures all the information about ${\mathcal {Q}}$ that is relevant for determining whether a given prefix intersecting ${\mathcal {Q}}$ is a minimal obstruction or not. Note that any given value of $size ({\mathcal {R}})$ can be present in only one triple from the deficiency pattern of ${\mathcal {Q}}$, because $def ({\mathcal {Q}}\cap {\mathcal {R}})$ and $I({\mathcal {Q}}\cap {\mathcal {R}}) \cap \delta (Q)$ only depend on $size ({\mathcal {R}})$ and ${\mathcal {Q}}$. See Fig. 5 for an example.

For an intuitive understanding of the role of deficiency patterns, consider a prefix ${\mathcal {T}}$ and a partial solution *U* for ${\mathcal {T}}$. In our algorithm, after we have decided on deleting *U*, we will not delete any further items from $I({\mathcal {T}})$; hence, it should not matter which items we have included in *U*, as long as its deletion leaves us with *the same kind of prefix*. So suppose that ${\mathcal {T}}'$ is a prefix that may or may not become a minimal obstruction after deleting *U*; clearly we may suppose that ${\mathcal {R}}={\mathcal {T}}'-U$ has a straight or a slant shape (otherwise it is certainly not a minimal obstruction).

In case ${\mathcal {T}}'-U$ contains ${\mathcal {T}}-U$, the only important properties of *U* are its size and its intersection with the boundary of ${\mathcal {T}}$: deleting any partial solution $U'$ with $|U|=|U'|$ that contains the same items from $\delta ({\mathcal {T}})$ as *U* will leave us with the same number of slots and the same number of items as the deletion of *U*. (See also Fig. 4 for an example showing why the boundary matters.)

In case ${\mathcal {T}}'-U={\mathcal {R}}$ does not contain ${\mathcal {T}}-U$ but intersects it, all further information necessary to “classify” *U* is contained in $defpat ({\mathcal {T}}-U)$. Indeed, to calculate the number of items in ${\mathcal {R}}$, it suffices to know the number of items in the intersection ${\mathcal {R}}\cap ({\mathcal {T}}-U)$ and the number of items that are contained in $I({\mathcal {R}}) \setminus I({\mathcal {T}}-U)$. The former can be calculated from the deficiency of ${\mathcal {R}}\cap ({\mathcal {T}}-U)$. For the latter we also need to know which items, among those occurring at positions of ${\mathcal {R}}$ outside ${\mathcal {T}}-U$, occur also in ${\mathcal {R}}\cap ({\mathcal {T}}-U)$; since such items are necessarily contained in the boundary of ${\mathcal {T}}$, it suffices to know the set $I(R \cap ({\mathcal {T}}-U)) \cap \delta ({\mathcal {T}})$.

**Strong equivalence** To formalize the above ideas, we call partial solutions $U_1$ and $U_2$ for ${\mathcal {T}}$ *strongly equivalent*, if

1. $$|U_1|=|U_2|$$,
2. $$U_1 \cap \delta ({\mathcal {T}})=U_2 \cap \delta ({\mathcal {T}})$$, and
3. $$defpat ({\mathcal {T}}-U_1)=defpat ({\mathcal {T}}-U_2)$$.

As the name suggests, strong equivalence is a sufficient condition for equivalence.

#### Lemma 6

If $U_1$ and $U_2$ are strongly equivalent partial solutions for ${\mathcal {T}}$, then they are equivalent as well.

#### Proof

Suppose that *W* is an extension for $({\mathcal {T}},U_1)$. We need to prove that it is an extension for $({\mathcal {T}},U_2)$ as well. Clearly, we have $W \subseteq I \setminus I({\mathcal {T}})$, so it suffices to show that ${\mathcal {P}}-(U_2 \cup W)$ is solvable.

Suppose for contradiction that ${\mathcal {Q}}_2$ is a minimal obstruction in ${\mathcal {P}}-(U_2 \cup W)$. Let us consider the prefix ${\mathcal {Q}}_1$ of ${\mathcal {P}}-(U_1 \cup W)$ that has the same size as ${\mathcal {Q}}_2$; such a prefix exists because $|U_1|=|U_2|$. In the remainder of the proof, we argue that ${\mathcal {Q}}_1$ is not solvable in ${\mathcal {P}}-(U_1 \cup W)$, contradicting the assumption that *W* is an extension for $({\mathcal {T}},U_1)$ and thus $U_1 \cup W$ is a solution for ${\mathcal {P}}$. Note that ${\mathcal {Q}}_1$ and ${\mathcal {Q}}_2$ clearly have the same slots, all of them complete.

Recall that $U_2$ is a partial solution for ${\mathcal {T}}$, so ${\mathcal {T}}-U_2$ is solvable. Since *W* is disjoint from $I({\mathcal {T}})$, we know that ${\mathcal {Q}}_2$ cannot be contained in ${\mathcal {T}}-U_2$.

First, let us assume that ${\mathcal {Q}}_2$ contains ${\mathcal {T}}-U_2$. In this case, $|U_1|=|U_2|$ and $U_1 \cap \delta ({\mathcal {T}})=U_2 \cap \delta ({\mathcal {T}})$ together immediately imply that ${\mathcal {Q}}_1$ and ${\mathcal {Q}}_2$ contain the same number of items: $|I({\mathcal {Q}}_1)|=|I({\mathcal {Q}}_2)|$. Hence, we get $|I({\mathcal {Q}}_1)|=|I({\mathcal {Q}}_2)|<|S({\mathcal {Q}}_2)|=|S({\mathcal {Q}}_1)|$, proving our claim.

Second, let us assume now that ${\mathcal {Q}}_2$ and ${\mathcal {T}}-U_2$ are intersecting, and let their intersection be ${\mathcal {T}}_2^{\cap }$. Similarly, let ${\mathcal {T}}_1^{\cap }$ be the intersection of ${\mathcal {Q}}_1$ and ${\mathcal {T}}-U_1$. Since ${\mathcal {Q}}_2$ is a minimal obstruction and thus has a straight or a slant shape, we know that $(size ({\mathcal {Q}}_2),def ({\mathcal {T}}_2^{\cap }),I({\mathcal {T}}_2^{\cap }) \cap \delta ({\mathcal {T}}-U_2))$ is contained in the deficiency pattern of ${\mathcal {T}}-U_2$. By the third condition of equivalence, the same triple must also be present in the deficiency pattern of ${\mathcal {T}}-U_1$. Hence, ${\mathcal {T}}_1^{\cap }$ must have the same deficiency as ${\mathcal {T}}_2^{\cap }$. By $|U_1|=|U_2|$ and $U_1 \cap \delta ({\mathcal {T}})=U_2 \cap \delta ({\mathcal {T}})$, we know that ${\mathcal {T}}-U_1$ and ${\mathcal {T}}-U_2$ have the same size, and thus ${\mathcal {T}}_1^{\cap }$ has the same size as ${\mathcal {T}}_2^{\cap }$. This implies $|I({\mathcal {T}}_1^{\cap })|=|I({\mathcal {T}}_2^{\cap })|$.

Moreover, we also get $I({\mathcal {T}}_2^{\cap }) \cap \delta ({\mathcal {T}}-U_2)=I({\mathcal {T}}_1^{\cap }) \cap \delta ({\mathcal {T}}-U_1)$. Recall that $U_1 \cap \delta ({\mathcal {T}}) = U_2 \cap \delta ({\mathcal {T}})$ is guaranteed by the second condition of strong equivalence. Hence, adding the items contained in ${\mathcal {Q}}_2$ but not in ${\mathcal {T}}$ increases the size of $I({\mathcal {T}}_2^{\cap })$ exactly as adding the items contained in ${\mathcal {Q}}_1$ but not in ${\mathcal {T}}$ increases the size of $I({\mathcal {T}}_1^{\cap })$. Therefore, we can conclude that $|I({\mathcal {Q}}_1)|=|I({\mathcal {Q}}_2)|$, which again implies that ${\mathcal {Q}}_1$ is not solvable. $\square$

Before giving the details of algorithm $$Extend $$, we need one more lemma on the relation of prefixes that we consider during the iterative approach of addressing minimal obstructions one-by-one.

#### Lemma 7

Let ${\mathcal {Q}}_0$ be a minimal obstruction in ${\mathcal {P}}-U_0$ for a set $U_0 \subseteq I$ of items, and let ${\mathcal {T}}$ be the largest^4^ prefix in ${\mathcal {P}}$ for which ${\mathcal {T}}-U_0={\mathcal {Q}}_0$. Let also $Y \subseteq I({\mathcal {T}})\setminus U_0$ with $1 \le |Y|\le 2$ be a partial solution for ${\mathcal {T}}-U_0$; then $U=U_0 \cup Y$ is a partial solution for ${\mathcal {T}}$. Now, let ${\mathcal {Q}}$ be a minimal obstruction in ${\mathcal {P}}-U$, and let ${\mathcal {T}}'$ be the largest prefix of ${\mathcal {P}}$ such that ${\mathcal {T}}'-U={\mathcal {Q}}$. Then either $I({\mathcal {T}}') \supseteq I({\mathcal {T}})$, or there does not exist an extension for $({\mathcal {T}},U)$.

**Fig. 6:**
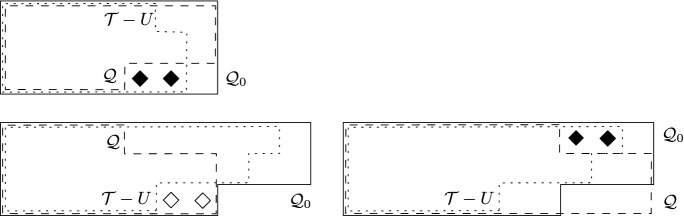
Illustration for the proof of Lemma 7. The shapes of prefixes ${\mathcal {Q}}_0$, ${\mathcal {Q}}$, and ${\mathcal {T}}-U={\mathcal {Q}}_0-Y$ are depicted with solid, dashed, and dotted lines, respectively. Positions contained in ${\mathcal {T}}-U$ but not in ${\mathcal {Q}}$ are marked by black diamonds, while positions contained in ${\mathcal {Q}}$ but not in ${\mathcal {T}}-U$ are marked by white diamonds

#### Proof

First observe that ${\mathcal {T}}-U$ cannot contain ${\mathcal {T}}'-U$, because ${\mathcal {T}}-U$ is solvable, but ${\mathcal {T}}'-U$ is a minimal obstruction. Assume now that $I({\mathcal {T}}') \not \supseteq I({\mathcal {T}})$. Then clearly, ${\mathcal {T}}'-U$ cannot contain ${\mathcal {T}}-U$ either. Hence, ${\mathcal {T}}-U$ must intersect with the minimal obstruction ${\mathcal {Q}}={\mathcal {T}}'-U$.

Consider agents’ preference lists in the profile ${\mathcal {P}}-U$ and the underlying graph $G_{{\mathcal {P}}-U}$. We say that two (or three) positions in the preference lists *belong* to the same slot in $G_{{\mathcal {P}}-U}$, if they are contained in $L_{I \setminus U}^x[i-2:i]$ for some agent *x* and index $i \equiv 1 \mod 3$. We claim that either

(i) all positions contained in ${\mathcal {T}}-U$ but not in ${\mathcal {Q}}$ belong to the same slot in $G_{{\mathcal {P}}-U}$, or conversely,
(ii) all positions contained in ${\mathcal {Q}}$ but not in ${\mathcal {T}}-U$ belong to the same slot in $G_{{\mathcal {P}}-U}$.

This claim can be seen by the heavy use of Lemma 2, distinguishing between cases depending on the shapes and positions of ${\mathcal {Q}}$ and ${\mathcal {Q}}_0$; see Fig. 6.

Let us first assume that ${\mathcal {Q}}_0$ has a straight shape, so ${\mathcal {Q}}_0=({\mathcal {P}}-U_0)[i,i,i]$ for some index $i \equiv 1 \mod 3$. Then by $|Y| \le 2$ we know that ${\mathcal {Q}}_0-Y={\mathcal {T}}-U$ contains $({\mathcal {P}}-U)[i-3,i-3,i-3]$ and is contained in $({\mathcal {P}}-U)[i,i,i]$. Hence, the only possible way for ${\mathcal {T}}-U$ to intersect ${\mathcal {Q}}$ is for ${\mathcal {Q}}$ to have size $[i,i,i-3]$, $[i,i-3,i]$, or $[i-3,i,i]$; w.l.o.g. we suppose ${\mathcal {Q}}=({\mathcal {P}}-U)[i,i,i-3]$. But then (i) must hold, because any position contained in ${\mathcal {T}}-U$ but not in ${\mathcal {Q}}$ must be a position in $L_{I \setminus U}^c[i-2,i]$.

Let us now assume that ${\mathcal {Q}}_0$ has a slant shape; w.l.o.g. we may assume that ${\mathcal {Q}}_0=({\mathcal {P}}-U_0)[i,i,i-3]$ for some index $i \equiv 1 \mod 3$. Now, if ${\mathcal {Q}}$ is contained in $({\mathcal {P}}-U)[i-3,i-3,i-3]$, then (ii) holds, because any position contained in ${\mathcal {Q}}$ but not in $Q_0-Y={\mathcal {T}}-U$ must be a position in $L_{I \setminus U}^c[i-4,i-3]$. Otherwise, using again $|Y|\le 2$, the only way for ${\mathcal {Q}}$ to intersect ${\mathcal {T}}-U$ is for ${\mathcal {Q}}$ to have size $[i-3,i,i]$ or $[i,i-3,i]$, and in either case (i) holds. This proves our claim.

Suppose now (i). Let (*x*, *j*) be the slot to which all positions contained in ${\mathcal {T}}-U$ but not in ${\mathcal {Q}}$ belong; let $I^\star$ denote the set of items on these positions in ${\mathcal {T}}-U$. Then

2 $$\begin{aligned} I({\mathcal {T}}-U) \setminus I({\mathcal {Q}})\subseteq I^\star . \end{aligned}$$

Since $I^\star$ contains items of ${\mathcal {T}}-U=Q_0-Y$, we know that they occur on positions belonging to the slot (*x*, *j*) in ${\mathcal {Q}}_0$ as well (note that $Q_0$ is a prefix in ${\mathcal {P}}-(U \setminus Y)$ not in ${\mathcal {P}}-U$). Thus $(x,j) \in S({\mathcal {Q}}_0)$, but observe that $(x,j+1) \notin S({\mathcal {Q}}_0)$. However, by the minimality of ${\mathcal {Q}}_0$, any item present in a last slot of ${\mathcal {Q}}_0$ occurs at least once more in ${\mathcal {Q}}_0$ (since an item eligible only for (*x*, *j*) among all slots in $S({\mathcal {Q}}_0)$ would imply that deleting the positions corresponding to (*x*, *j*) from ${\mathcal {Q}}_0$ would yield a prefix with $|S({\mathcal {Q}}_0)|-1$ slots and at most $|I({\mathcal {Q}}_0)|-1$ items). Therefore, any item of $I^\star$ occurs at least once more in ${\mathcal {Q}}_0$. By $I^\star \cap Y=\emptyset$, this implies that any item of $I^\star$ occurs at least once in a position of ${\mathcal {Q}}_0-Y={\mathcal {T}}-U$ that does not belong to (*x*, *j*). However, any such position is contained in ${\mathcal {Q}}$ as well, by our assumption (i). Thus $I^\star \subseteq I({\mathcal {Q}})$, which by Equality (2) implies $\emptyset =I({\mathcal {T}}-U) \setminus I({\mathcal {Q}})=I({\mathcal {T}}-U) \setminus I({\mathcal {T}}'-U)$. This proves $I({\mathcal {T}}) \subseteq I({\mathcal {T}}')$.

Supposing (ii), let (*x*, *j*) be the slot to which all positions contained in ${\mathcal {Q}}$ but not in ${\mathcal {T}}-U$ belong; let $I^\star$ denote the set of items on these positions in ${\mathcal {Q}}$. Then it is clear that $(x,j) \in S({\mathcal {Q}})$ but $(x,j+1)\notin S({\mathcal {Q}})$, hence arguing as above, we get that $I^\star \subseteq I({\mathcal {T}}-U)$. But then $I({\mathcal {Q}}) \subseteq I({\mathcal {T}}-U) \cup I^\star \subseteq I({\mathcal {T}}-U)$, implying that the minimal obstruction ${\mathcal {Q}}$ in ${\mathcal {P}}-U$ contains only items that are forbidden w.r.t. $({\mathcal {T}},U)$. Thus, there cannot exist an extension for $({\mathcal {T}},U)$. $\square$

Now, we are ready to describe algorithm $$Extend $$ in detail. Let $$({\mathcal {T}},U)$$ be the input for $$Extend $$. Throughout the run of the algorithm, we will store all inputs with which $$Extend $$ has been computed in a table $$SolTable $$, keeping track of the corresponding extensions as well. Initially, we call $$Extend $$ with input $$({\mathcal {T}}_{\emptyset },\emptyset )$$, where $${\mathcal {T}}_{\emptyset }$$ denotes the empty prefix of our input profile $${\mathcal {P}}$$, i.e., $${\mathcal {P}}[0,0,0]$$, and we initialize $$SolTable $$ as empty. For an example of running algorithm $$Extend $$ on an instance of PID, see Appendix A.

**Algorithm **$$Extend ({\mathcal {T}},U)$$:

**Step 0:** **Check for strongly equivalent inputs.**
  For each $$({\mathcal {T}},U')$$ in $$SolTable $$, check whether $$U'$$ and *U* are strongly equivalent with respect to $${\mathcal {T}}$$, and if so, return $$Extend ({\mathcal {T}},U')$$.
**Step 1:** **Check for trivial solution.**
  Check if $${\mathcal {P}}-U$$ is solvable. If so, then return the empty extension $$\emptyset $$, and store the entry $$({\mathcal {T}},U)$$ together with the value $$\emptyset $$ in $$SolTable $$.
**Step 2:** **Find a minimal obstruction.**
  Find a minimal obstruction ${\mathcal {Q}}$ in ${\mathcal {P}}-U$; recall that ${\mathcal {P}}-U$ is not solvable in this step.
**Step 3:** **Compute the new prefix.**
  Let $${\mathcal {T}}'$$ be the largest prefix of $${\mathcal {P}}$$ for which $${\mathcal {T}}'-U={\mathcal {Q}}$$. If $$I({\mathcal {T}}') \not \supseteq I({\mathcal {T}})$$, then return ‘No’, and store the entry $$({\mathcal {T}},U)$$ together with the value ‘No’ in $$SolTable $$.
**Step 4:** **Compute a branching set.**
  Using Lemma 5, determine a branching set $${\mathcal {Y}}$$ for $${\mathcal {Q}}$$ forbidding $$I({\mathcal {T}}) \setminus U$$. If $${\mathcal {Y}}=\emptyset $$, then return ‘No’, and store the entry $$({\mathcal {T}},U)$$ together with the value ‘No’ in $$SolTable $$.
**Step 5:** **Branch.**
  For each $Y \subseteq {\mathcal {Y}}$, compute $E_Y:=Extend ({\mathcal {T}}',U \cup Y)$.
**Step 6:** **Find a smallest extension.**
  Compute a set $$E_{Y^\star }$$ for which $$|Y^\star \cup E_{Y^\star }|=\min _{Y \in {\mathcal {Y}}} |Y \cup E_Y|$$. Return the set $$Y^\star \cup E_{Y^\star }$$, and store the entry $$({\mathcal {T}},U)$$ together with the extension $$Y^\star \cup E_{Y^\star }$$ in $$SolTable $$.

#### Lemma 8

When initially called with input $$({\mathcal {T}}_{\emptyset },\emptyset )$$, algorithm $$Extend $$ is correct, i.e., for any prefix $${\mathcal {T}}$$ of $${\mathcal {P}}$$ and any partial solution *U* for $${\mathcal {T}}$$, $$Extend ({\mathcal {T}},U)$$ returns a minimum-size extension for $$({\mathcal {T}},U)$$ (if existent).

#### Proof

Observe that it suffices to prove the claim for those cases when algorithm $$Extend $$ does not return a solution in Step 0: its correctness in the remaining cases (so when a solution contained in $$SolTable $$ for a strongly equivalent input is found and returned in Step 0) follows from Lemma 6.

We are going to prove the lemma by induction on $$|I \setminus U|$$. Clearly, if $$|I \setminus U|=0$$, then $${\mathcal {P}}-U$$ is an empty instance, and hence is trivially solvable. Assume now that $$I \setminus U \ne \emptyset $$, and that $$Extend $$ returns a correct output for any input $$({\mathcal {T}}_0,U_0)$$ with $$|I \setminus U_0|<|I \setminus U|$$.

First, if the algorithm returns $\emptyset$ in Step 1, then this is clearly correct.

Second, if it returns ‘No’ in Step 3, then in this case ${\mathcal {T}}\ne {\mathcal {T}}_{\emptyset }$, so $Extend ({\mathcal {T}},U)$ is a recursive call, and hence was called when branching on a branching set. Thus, there exists some $Y \subseteq U$ with $1 \le |Y| \le 2$ for which ${\mathcal {T}}-(U \setminus Y)$ is a minimal obstruction $Q_0$. Hence, Lemma 7 can be applied, which implies the correctness of this step.

Third, if the algorithm returns ‘No’ in Step 4 because it finds that the branching set ${\mathcal {Y}}$ forbidding $I({\mathcal {T}}) \setminus U$ for the minimal obstruction ${\mathcal {Q}}$ is empty, then this, by the definition of a branching set (forbidding $I({\mathcal {T}}) \setminus U$) and by the soundness of the algorithm of Lemma 5, means that there is no solution *S* for ${\mathcal {P}}-U$ disjoint from $I({\mathcal {T}}) \setminus U$. But then we also know that there is no solution *S* for ${\mathcal {P}}$ for which $S \cap I({\mathcal {T}})=U$ holds, so there is no extension for $({\mathcal {T}},U)$. Hence this step is correct as well.

Therefore, we can assume that the algorithm’s output is $$Y^\star \cup E_{Y^\star }$$ for some $$Y^\star \in {\mathcal {Y}}$$, where $$E_{Y^\star }=Extend ({\mathcal {T}}', U \cup Y^\star )$$ and $${\mathcal {T}}'$$ is the largest profile in $${\mathcal {P}}$$ for which $${\mathcal {T}}'-U={\mathcal {Q}}$$. As $$|I \setminus (U \cup Y)| < |I \setminus U|$$ for any $$Y \in {\mathcal {Y}}$$, the induction hypothesis implies that $$Extend $$ runs correctly on all inputs $$({\mathcal {T}}', U \cup Y)$$, $$Y \in {\mathcal {Y}}$$. Hence, $$E_{Y^\star }$$ is an extension for $$({\mathcal {T}}',U \cup Y^\star )$$ and so $$U \cup Y^\star \cup E_{Y^\star }$$ is a solution for $${\mathcal {P}}$$. Moreover, since $$E_{Y^\star } \cap I({\mathcal {T}}') = \emptyset $$ and $$I({\mathcal {T}}') \supseteq I({\mathcal {T}})$$ by Step 3, we know that $$E_{Y^\star }$$ is disjoint from $$I({\mathcal {T}})$$. Since $$Y^\star $$ is contained in a branching set for $${\mathcal {Q}}$$ in $${\mathcal {P}}-U$$ forbidding $$I({\mathcal {T}}) \setminus U$$, we get that $$Y^\star \cup E_{Y^\star }$$ is disjoint from $$I({\mathcal {T}})$$ as well. Thus, $$Y^\star \cup E_{Y^\star }$$ is an extension for $$({\mathcal {T}},U)$$.

It remains to argue that if *E* is an extension for $({\mathcal {T}},U)$, then $|Y^\star \cup E_{Y^\star }| \le |E|$. Clearly, *E* is a solution for ${\mathcal {P}}-U$ disjoint from $I({\mathcal {T}}) \setminus U$. By the definition of a branching set forbidding $I({\mathcal {T}}) \setminus U$ and the correctness of Lemma 5, we know that there must exist a solution $E'$ for ${\mathcal {P}}-U$ disjoint from $I({\mathcal {T}}) \setminus U$ and having size $|E'| \le |E|$ for which $E' \cap I({\mathcal {Q}}) \in {\mathcal {Y}}$. Define $Y'=E' \cap I({\mathcal {Q}})$. Observe that $E' \setminus Y'$ an extension for $({\mathcal {T}}',U \cup Y')$.

Using again the induction hypothesis, we get that $$Extend ({\mathcal {T}}',U \cup Y')$$ returns an extension $$E_{Y'}$$ for $$({\mathcal {T}}',U \cup Y')$$ of minimum size, so in particular, $$|E_{Y'}| \le |E' \setminus Y'|$$, implying $$|Y' \cup E_{Y'}| \le |E'| \le |E|$$. Thus, by our choice of $$Y^\star $$, we get that the output of $$Extend ({\mathcal {T}},U)$$ (that is, $$Y^\star \cup E_{Y^\star }$$) has size at most |*E*|. This proves our claim. Therefore, we get that if $$Extend $$ returns an output in Step 6, then this output is correct. $$\square $$

Lemma 8 immediately gives us an algorithm to solve PID: $Extend ({\mathcal {T}}_{\emptyset },\emptyset )$ returns a solution *S* for ${\mathcal {P}}$ of minimum size; we only have to compare |*S*| with the desired solution size *k*.

The next lemma states that $$Extend $$ gets called polynomially many times.

#### Lemma 9

Throughout the run of algorithm $$Extend $$ initially called with input $$({\mathcal {T}}_{\emptyset },\emptyset )$$, the table $$SolTable $$ contains $$O(|I|^{7})$$ entries.

#### Proof

Let us consider table $$SolTable $$ at a given moment during the course of algorithm $$Extend $$, initially called with the input $$({\mathcal {T}}_{\emptyset },\emptyset )$$ (and having possibly performed several recursive calls since then). Let us fix a prefix $${\mathcal {T}}$$. We are going to give an upper bound on the maximum cardinality of the family $${\mathcal {U}}_{{\mathcal {T}}}$$ of partial solutions *U* for $${\mathcal {T}}$$ for which $$SolTable $$ contains the entry $$({\mathcal {T}},U)$$.

By Step 0 of algorithm $$Extend $$, no two sets in $${\mathcal {U}}_{{\mathcal {T}}}$$ are strongly equivalent. Recall that if $$U_1$$ and $$U_2$$, both in $${\mathcal {U}}_{{\mathcal {T}}}$$, are not strongly equivalent with respect to $${\mathcal {T}}$$, then either $$|U_1| \ne |U_2|$$, or $$\delta ({\mathcal {T}}) \cap U_1 \ne \delta ({\mathcal {T}}) \cap U_2$$, or $$defpat ({\mathcal {T}}-U_1)\ne defpat ({\mathcal {T}}-U_2)$$. Let us partition the sets in $${\mathcal {U}}_{{\mathcal {T}}}$$ into *groups*: we put $$U_1$$ and $$U_2$$ in the same group, if $$|U_1|=|U_2|$$ and $$\delta ({\mathcal {T}}) \cap U_1 = \delta ({\mathcal {T}}) \cap U_2$$.

Examining Steps 2–4 of algorithm $$Extend $$, we can observe that if $$U \ne \emptyset $$, then for some $$Y_U \subseteq U$$ of size 1 or 2, the prefix $${\mathcal {T}}-(U \setminus Y_U)$$ is a minimal obstruction $${\mathcal {Q}}_U$$. Since removing items from a prefix cannot increase the size of its boundary, Lemma 3 implies that the boundary of $${\mathcal {T}}-U$$ contains at most 3 items. We get $$|\delta ({\mathcal {T}}) \setminus U| = |\delta ({\mathcal {T}}-U)| \le 3$$, from which it follows that $$\delta ({\mathcal {T}}) \cap U$$ is a subset of $$\delta ({\mathcal {T}})$$ of size at least $$|\delta ({\mathcal {T}})|-3$$. Therefore, the number of different values that $$\delta ({\mathcal {T}}) \cap U$$ can take is $$O(|I|^3)$$. Since any $$U \in {\mathcal {U}}_{{\mathcal {T}}}$$ has size at most |*I*|, we get that there are $$O(|I|^4)$$ groups in $${\mathcal {U}}_{{\mathcal {T}}}$$. Let us fix some group $${\mathcal {U}}_g$$ of $${\mathcal {U}}_{{\mathcal {T}}}$$. We are going to show that the number of different deficiency patterns for $${\mathcal {T}}-U$$ where $$U \in {\mathcal {U}}_g$$ is constant.

Recall that the deficiency pattern of ${\mathcal {T}}-U$ consists of triples of the form

$$\begin{aligned} (size ({\mathcal {R}}),def ({\mathcal {R}}^{\cap }),I({\mathcal {R}}^{\cap }) \cap \delta ({\mathcal {T}}-U)) \end{aligned}$$

where ${\mathcal {R}}$ is some prefix of ${\mathcal {P}}-U$ with a slant or a straight shape, and ${\mathcal {R}}^{\cap }$ is the intersection of ${\mathcal {T}}-U$ and ${\mathcal {R}}$.

First observe that by the definition of a group, $size ({\mathcal {T}}-U_1)=size ({\mathcal {T}}-U_2)$ holds for any $U_1, U_2 \in {\mathcal {U}}_g$. Let us fix an arbitrary $U \in {\mathcal {U}}_g$. Since ${\mathcal {T}}-U$ can be obtained by deleting 1 or 2 items from a minimal obstruction, Lemma 2 implies that there can only be a constant number of prefixes ${\mathcal {R}}$ of ${\mathcal {P}}-U$ which intersect ${\mathcal {T}}-U$ and have a slant or a straight shape; in fact, it is not hard to check that the number of such prefixes ${\mathcal {R}}$ is at most 5 for any given ${\mathcal {T}}-U$. Therefore, the number of values taken by the first coordinate $size ({\mathcal {R}})$ of any triple in the deficiency pattern of ${\mathcal {T}}-U$ is constant. Since ${\mathcal {T}}-U$ has the same size for any $U \in {\mathcal {U}}_g$, we also get that these values coincide for any $U \in U_g$. Hence, we obtain that (A) the total number of values the first coordinate of any triple in the deficiency pattern of ${\mathcal {T}}-U$ for any $U \in {\mathcal {U}}_g$ can take is constant.

Let ${\mathcal {R}}^{\cap }$ be the intersection of ${\mathcal {T}}-U$ and some prefix of straight or slant shape. By definition, ${\mathcal {R}}^{\cap }$ is contained in ${\mathcal {Q}}_U$. By $|Y_U| \le 2$, there are only a constant number of positions which are contained in ${\mathcal {Q}}_U$ but not in ${\mathcal {R}}^{\cap }$. From this both $||I({\mathcal {R}}^{\cap })| - |I({\mathcal {Q}}_U)||=O(1)$ and $||S({\mathcal {R}}^{\cap })| - |S({\mathcal {Q}}_U)||=O(1)$ follow. As ${\mathcal {Q}}_U$ is a minimal obstruction, we also have $|I({\mathcal {Q}}_U)|=|S({\mathcal {Q}}_U)|-1$, implying that (B) the deficiency $def ({\mathcal {R}}^{\cap })= |S({\mathcal {R}}^{\cap })|-|I({\mathcal {R}}^{\cap })|$ can only take a constant number of values too; note that we have an upper bound on $|def ({\mathcal {R}}^{\cap })|$ that holds for any $U \in {\mathcal {U}}_g$. Considering that $I({\mathcal {R}}^{\cap }) \cap \delta ({\mathcal {T}}-U)$ is the subset of $\delta ({\mathcal {T}}-U)$, and we also know $|\delta ({\mathcal {T}}-U)| \le 3$, we obtain that (C) the set $I({\mathcal {R}}^{\cap }) \cap \delta ({\mathcal {T}}-U)$ can take at most $2^3$ values (again, for all $U \in U_g$).

Putting together observations (A), (B), and (C), it follows that the number of different deficiency patterns of $${\mathcal {T}}-U$$ taken over all $$U \in {\mathcal {U}}_{g}$$ is constant. This implies $$|{\mathcal {U}}_{{\mathcal {T}}}|=O(|I|^4)$$. Since there are $$O(|I|^3)$$ prefixes $${\mathcal {T}}$$ of $${\mathcal {P}}$$, we arrive at the conclusion that the maximum number of entries in $$SolTable $$ is $$O(|I|^7)$$. $$\square $$

We are now able to formulate our main theorem, stating that PID is solvable in polynomial time for three agents.

#### Theorem 7

Proportional Item Deletions for three agents can be solved in time $O(|I|^{11})$.

#### Proof

By Lemma 8, we know that algorithm $Extend ({\mathcal {T}}_{\emptyset },\emptyset )$ returns a solution for ${\mathcal {P}}$ of minimum size, solving PID.

To bound the running time of $$Extend ({\mathcal {T}}_{\emptyset },\emptyset )$$, let us first give a bound on the time necessary for the computations performed by $$Extend $$ on some input $$({\mathcal {T}},U)$$ when not counting the computations performed in recursive calls. Clearly, Step 0 takes *O*(|*I*|) time (assuming we can effectively search within the table $$SolTable $$). Steps 1 and 2 can be accomplished in $$O(|I|^3)$$ time, as described in Lemma 1. Step 3 can be accomplished in time *O*(|*I*|). Using Lemma 5, Step 4 can be performed in $$O(|I|^4)$$ time. Since the cardinality of the branching set found in Step 4 is $$O(|I|^2)$$, Steps 5 and 6 can be performed in $$O(|I|^3)$$ time. Hence, any call to $$Extend $$ can be performed in $$(|I|^4)$$ time (when not counting the computations performed in the recursive calls).

Let us distinguish now between two types of recursive calls to $$Extend $$: a call $$Extend ({\mathcal {T}},U)$$ is *regular*, if Step 0 does *not* produce an output during its execution; otherwise we refer to this call as a *shadow* call. We first give an upper bound for the time spent on regular calls. Note that in each such call, an entry is added to $$SolTable $$. By Lemma 9, $$SolTable $$ contains $$O(|I|^7)$$ entries, and therefore the number of regular calls to $$Extend $$ is also $$O(|I|^7)$$. This gives us an upper bound of $$O(|I|^{11})$$ on the total time spent on regular calls to $$Extend $$.

To bound the time spent on shadow calls, observe that each regular call may give rise to at most $O(|I|^2)$ shadow calls (and there are no recursive calls performed within a shadow call). Hence, the number of shadow calls is $O(|I|^9)$. Since Step 0 takes *O*(|*I*|) time, this yields a bound of $O(|I|^{10})$ for the total time spent on shadow calls. Hence the total running time of $Extend ({\mathcal {T}}_{\emptyset },\emptyset )$ is as claimed. $\square$

## PID with Fixed Allocation

In this section we investigate a version of PID where an allocation is given in advance, and we want to make this allocation proportional by item deletion. The input of the problem, which we refer to as PID with Fixed Allocation, consists of a preference profile ${\mathcal {P}}=(N,I,L)$, an allocation $\pi :I \rightarrow N$, and an integer $k \in {\mathbb {N}}$. We call a set $S \subseteq I$ of items a *solution* for $({\mathcal {P}},\pi )$, if the restriction of $\pi$ to $I \setminus S$ is proportional for the profile ${\mathcal {P}}-S$; the task is to find a solution of size at most *k* for $({\mathcal {P}},\pi )$.

Since we are given a fixed allocation, the concept of minimal obstruction can be simplified accordingly: we say that agent *x* *becomes envious* at index *i* for some $i \in \{1,\dots ,|I|\}$, if the number of items in $L^x[1:i]$ assigned to *x* by $\pi$ is less than *i*/|*N*|, but for any smaller index $j<i$ the number of items in $L^x[1:j]$ assigned to *x* by $\pi$ is at least *j*/|*N*|. Clearly, if no agent becomes envious at any index, then our allocation $\pi$ is proportional.

Our first observation is that PID with Fixed Allocation can be solved in polynomial time if there are only two agents. To show this, we propose an algorithm that we call GreedyDel. Suppose that *N* contains only two agents. For an agent $x \in N$, we denote by ${\bar{x}}$ the other agent, i.e., ${\bar{x}} \in N$, $x \ne {\bar{x}}$.

**Algorithm** GreedyDel$$({\mathcal {P}},\pi ,k)$$:

**Step 0:** If $k<0$, then return ‘No’.
**Step 1:** If $\pi$ is proportional for ${\mathcal {P}}$, then return ‘Yes’.
**Step 2:** Perform a greedy deletion:
  Let *x* denote an agent and *i* an index such that *x* becomes envious at *i*. Compute the item *s* that is the least preferred by agent ${\bar{x}}$ among all items of $L^x[1:i]$ assigned to ${\bar{x}}$ by $\pi$, and call $GreedyDel ({\mathcal {P}}-\{s\}, {\left. \pi \right| _{I\setminus \{s\}}},k-1)$.

### Theorem 8

If the number of agents is two, then PID with Fixed Allocation can be solved in polynomial time by algorithm GreedyDel.

### Proof

It is easy to see that algorithm GreedyDel can be implemented in quadratic running time. To prove its correctness, let us consider the steps of GreedyDel on our input instance $({\mathcal {P}},\pi ,k)$.

Note that Steps 0 and 1 are clearly correct. We claim that the deletion performed in Step 2 is *safe* in the following sense: if *S* is a solution for $({\mathcal {P}},\pi )$ and *s* is the item deleted by the algorithm in Step 2, then there exists a solution $S'$ for $({\mathcal {P}},\pi )$ that contains *s*. Clearly, if this holds, then GreedyDel is correct.

Let *x* be the agent and *i* the index for which *x* becomes envious at *i*, as found in Step 2. Let $S_{[1:i]}$ denote those items of *S* that are contained in $L^x[1:i]$. Suppose that *S* does not contain the deleted item *s*. However, since *S* is a solution, we know that $S_{[1:i]}$ must contain strictly more items assigned by $\pi$ to ${\bar{x}}$ than to *x*, i.e.,

3 $$\begin{aligned} |S_{[1:i]} \cap \pi ^{-1}({\bar{x}})|\ge |S_{[1:i]} \cap \pi ^{-1}(x)|+1. \end{aligned}$$

Let $s^\star$ be the item least preferred by *x* in $S_{[1:i]} \cap \pi ^{-1}({\bar{x}})$. We will show that $S' = S \setminus \{s^\star \} \cup \{s\}$ is a solution for $({\mathcal {P}},\pi )$, proving our claim that Step 2 is safe.

Note that by our choice of *s*, agent ${\bar{x}}$ prefers $s^\star$ to *s*, so ${\bar{x}}$ cannot become envious at any index in ${\mathcal {P}}-S'$. If *x* prefers *s* to $s^\star$, then *x* cannot become envious at any index in ${\mathcal {P}}-S'$ either (because deleting an item assigned to ${\bar{x}}$ that comes before $s^\star$ in $L^x$ is always preferable for *x* than deleting $s^\star$), so in this case $S'$ is a solution for $({\mathcal {P}},\pi )$. Thus, for the sake of contradiction, assume that *x* also prefers $s^\star$ to *s*, and *x* becomes envious somewhere in ${\mathcal {P}}-S'$. This means that there exists an index $j \in \{1,\dots , |I|\}$ such that

4 $$\begin{aligned} |J_x \setminus S'| < |J_{{\bar{x}}} \setminus S'|, \end{aligned}$$

where $J_x$ and $J_{{\bar{x}}}$ denote the set of items in $L^x[1:j]$ assigned to *x* and to ${\bar{x}}$ by $\pi$, respectively (note that $L^x$ is the preference list of *x* in the original instance ${\mathcal {P}}$).

First, if $J_{{\bar{x}}}$ contains either both *s* and $s^\star$ or neither of them, then $|J_{{\bar{x}}} \cap S|=|J_{{\bar{x}}} \cap S'|$, so

$$\begin{aligned} |J_x \setminus S'|=|J_x \setminus S| \ge |J_{{\bar{x}}} \setminus S|=|J_{{\bar{x}}}\setminus S'|, \end{aligned}$$

where the first equality is implied by $\{s,s^\star \} \cap J_x= \emptyset$, and the inequality follows from the fact that *S* is a solution for $({\mathcal {P}},\pi )$. This contradicts (4).

Second, if $|J_{{\bar{x}}} \cap \{s,s^\star \}|=1$, then $s^\star \in J_{{\bar{x}}}$ but $s \notin J_{{\bar{x}}}$, because *x* prefers $s^\star$ to *s*. In this case we know $j<i$. Using that $s^\star$ is the least preferred by *x* among all items of $S_{[1:i]} \cap \pi ^{-1}({\bar{x}})$ but it still falls within $L^x[1:j]$, we know that *all* items of $S_{[1:i]} \cap \pi ^{-1}({\bar{x}})$ fall within $L^x[1:j]$ and are thus contained in $J_{{\bar{x}}}$. From this, we get that

$$\begin{aligned} |J_{{\bar{x}}} \cap S'|= & {} |J_{{\bar{x}}} \cap S|-1=|S_{[1:i]} \cap \pi ^{-1}({\bar{x}})|-1 \ge |S_{[1:i]} \cap \pi ^{-1}(x)| \ge |J_x \cap S|\\= & {} |J_x \cap S'|, \end{aligned}$$

where the first inequality follows from (3). Recall that *x* only becomes envious at *i* in ${\mathcal {P}}$, and therefore $|J_x|\ge |J_{{\bar{x}}}|$, leading us to

$$\begin{aligned} |J_x \setminus S'|=|J_x|-|J_x \cap S'| \ge |J_{{\bar{x}}}|-|J_{{\bar{x}}} \cap S'|=|J_{{\bar{x}}} \setminus S'|, \end{aligned}$$

which again contradicts (4). $\square$

Interestingly, PID with Fixed Allocation becomes ${{\mathsf {N}}}{{\mathsf {P}}}$-hard if the number of agents is 6. Hence, providing an allocation in advance does not seem to make PID much easier; the intuitive argument behind this is that we may be given an allocation that is quite unreasonable and may actually become a hindrance to proportionality instead of helping us. Our results leave open the computational complexity of the problem when the number of agents is in $\{3,4,5\}$.

### Theorem 9

If the number of agents is six, then PID with Fixed Allocation is ${{\mathsf {N}}}{{\mathsf {P}}}$-complete.

### Proof

It is straightforward to see that the problem is in ${{\mathsf {N}}}{{\mathsf {P}}}$. To prove its ${{\mathsf {N}}}{{\mathsf {P}}}$-completeness, we are going to present a reduction from the Cubic Monotone 1-in-3-SAT problem, whose input is a propositional formula $\varphi$ in conjunctive normal form where variables only occur as positive literals, and the underlying graph *G* is a cubic graph (i.e., every vertex has degree 3). Formally, we define $G=(V \cup {\mathcal {C}},E)$ as a bipartite graph whose two partitions are the set *V* of variables and the set ${\mathcal {C}}$ of clauses, and a variable $v \in V$ is adjacent to a clause $C \in {\mathcal {C}}$ in *G* if and only if *v* appears in *C*. Since *G* is cubic, each variable occurs in exactly three clauses, and conversely, each clause contains exactly three distinct variables. The task in the Cubic Monotone 1-in-3-SAT problem is to decide whether there exists a truth assignment for $\varphi$ where exactly one out of three variables is true in each clause; we will call such a truth assignment *valid*. Moore and Robson [21] proved that this problem is ${{\mathsf {N}}}{{\mathsf {P}}}$-hard.

Given the input formula $\varphi$, we construct an instance $({\mathcal {P}},\pi ,k)$ of PID with Fixed Allocation such that $\varphi$ is satisfiable if and only if $({\mathcal {P}},\pi )$ admits a solution of size at most *k*. Let $\varphi$ contain variables $v_1,\dots ,v_n$ and clauses $C_1,\dots ,C_n$; note that $n \equiv 0 \mod 3$ must hold, as *G* is cubic. We define the set of agents as $N=\{v,v',w,w',f,f'\}$. The set *I* of items contains $V_i=\{a_i,b_i,c_i\}$ and ${\hat{V}}_i=\{{\hat{a}}_i,{\hat{b}}_i,{\hat{c}}_i\}$ for each variable $v_i \in V$, as well as a set $I_x$ containing some newly introduced items for each agent $x \in N$; we set $|I_f|=3n$, $|I_{f'}|=2n$, and $|I_x|=4n$ for any other agent $x \in N\setminus \{f,f'\}$. We let $U=V_1 \cup \dots \cup V_n$ and ${\hat{U}}={\hat{V}}_1 \cup \dots \cup {\hat{V}}_n$. Items in *U* and ${\hat{U}}$ are assigned by $\pi$ to $f'$ and *f*, respectively, while for any agent $x \in N$, items in $I_x$ are assigned to *x* by $\pi$. We set $k=3n$.

To finish the definition of profile ${\mathcal {P}}=(N,I,L)$, it remains to give the preferences of each agent, for which we need additional notation. The three items in $V_i$ will correspond to the three occurrences of variable $v_i$, each a positive literal, while the items in ${\hat{V}}_i$ will correspond to their negated forms. Hence, for each clause $C_i \in {\mathcal {C}}$, we define $P_i$ as the set of items that, for any $j \in \{1,\dots , n\}$, contains item $a_j$, $b_j$, or $c_j$ if and only if $C_i$ contains the first, second, or third occurrence, respectively, of variable $v_j$ in $\varphi$. We also define $N_i$ as the set of items containing ${\hat{a}}_j$, ${\hat{b}}_j$, or ${\hat{c}}_j$ for some *j* if and only if $P_i$ contains $a_j$, $b_j$, or $c_j$, respectively.

To simplify our notation for the preference lists, we fix an arbitrary ordering $\prec$ over *I* so that we can omit listing all irrelevant items in the preference lists: the symbol ‘’ at the end of a preference list stands for the sequence of all remaining items according to their order in $\prec$. Also, we write [*X*] for a set *X* of items to denote their sequence according to $\prec$. In the preference list of some agent $x \in N$, it will be sufficient to distinguish between items of $D=I_v \cup I_{v'} \cup I_w \cup I_{w'}$ only up to the point of indicating whether they are assigned to *x* by $\pi$ or not. Thus, we will use ‘$\bullet$’ symbols in $L^x$ to denote items from $I_x$, and we will use ‘$\circ$’ symbols to denote items from $D \setminus I_x$ (these will serve as dummy items). If the number of ‘$\bullet$’ (or ‘$\circ$’) symbols in $L^x$ is $\ell$, then they refer to the first $\ell$ items from $I_x$ (or from $D \setminus I_x$, respectively) according to $\prec$. Now, the preference lists are as shown in Table 1.

**Table 1:** Preferences in the instance of PID with Fixed Allocation constructed in the proof of Theorem 9.

$L^{v}:$	$\bullet ,\overbrace{\circ , \circ , \circ , \circ , a_1,{\hat{a}}_1}^{6},$	$\bullet ,\overbrace{\circ ,\circ , \circ , \circ , b_1, {\hat{b}}_1}^{6},$	$\bullet ,\overbrace{\circ , \circ , \circ , \circ , c_1, {\hat{c}}_1}^{6},$	
	$\bullet ,\circ , \circ , \circ , \circ , a_2, {\hat{a}}_2,$	$\bullet ,\circ , \circ , \circ , \circ , b_2, {\hat{b}}_2,$	$\bullet ,\circ , \circ , \circ , \circ , c_2, {\hat{c}}_2,$	
	$$\vdots $$			
	$\bullet ,\circ , \circ , \circ , \circ , a_n, {\hat{a}}_n,$	$\bullet ,\circ , \circ , \circ , \circ , b_n, {\hat{b}}_n,$	$\bullet ,\circ , \circ , \circ , \circ , c_n, {\hat{c}}_n,$	
$L^{v'}:$	$\bullet ,\circ , \circ , \circ , \circ , c_n, {\hat{c}}_n,$	$\bullet ,\circ , \circ , \circ , \circ , b_n, {\hat{b}}_n,$	$\bullet ,\circ , \circ , \circ , \circ , a_n, {\hat{a}}_n,$	
	$$\vdots $$			
	$\bullet ,\circ , \circ , \circ , \circ , c_2, {\hat{c}}_2,$	$\bullet ,\circ , \circ , \circ , \circ , b_2, {\hat{b}}_2,$	$\bullet ,\circ , \circ , \circ , \circ , a_2, {\hat{a}}_2,$	
	$\bullet ,\circ , \circ , \circ , \circ , c_1, {\hat{c}}_1,$	$\bullet ,\circ , \circ , \circ , \circ , b_1, {\hat{b}}_1,$	$\bullet ,\circ , \circ , \circ , \circ , a_1, {\hat{a}}_1,$	
$L^{w}:$	$\bullet ,\circ , \circ , \circ , \circ , {\hat{a}}_1, b_1,$	$\bullet ,\circ , \circ , \circ , \circ , {\hat{b}}_1, c_1,$	$\bullet ,\circ , \circ , \circ , \circ , {\hat{c}}_1, a_1,$	
	$\bullet ,\circ , \circ , \circ , \circ , {\hat{a}}_2, b_2,$	$\bullet ,\circ , \circ , \circ , \circ , {\hat{b}}_2, c_2,$	$\bullet ,\circ , \circ , \circ , \circ , {\hat{c}}_2, a_2,$	
	$$\vdots $$			
	$\bullet ,\circ , \circ , \circ , \circ , {\hat{a}}_n, b_n,$	$\bullet ,\circ , \circ , \circ , \circ , {\hat{b}}_n, c_n,$	$\bullet ,\circ , \circ , \circ , \circ , {\hat{c}}_n, a_n,$	
$L^{w'}:$	$\bullet ,\circ , \circ , \circ , \circ , {\hat{c}}_n, a_n,$	$\bullet ,\circ , \circ , \circ , \circ , {\hat{b}}_n, c_n,$	$\bullet ,\circ , \circ , \circ , \circ , {\hat{a}}_n, b_n,$	
	$$\vdots $$			
	$\bullet ,\circ , \circ , \circ , \circ , {\hat{c}}_2, a_2,$	$\bullet ,\circ , \circ , \circ , \circ , {\hat{b}}_2, c_2,$	$\bullet ,\circ , \circ , \circ , \circ , {\hat{a}}_2, b_2,$	
	$\bullet ,\circ , \circ , \circ , \circ , {\hat{c}}_1, a_1,$	$\bullet ,\circ , \circ , \circ , \circ , {\hat{b}}_1, c_1,$	$\bullet ,\circ , \circ , \circ , \circ , {\hat{a}}_1, b_1,$	
$L^{f}:$	$\bullet ,\overbrace{\circ , \circ , \circ ,[P_1]}^{6},$	$\bullet ,\overbrace{\circ ,\circ , \circ , [P_2]}^{6}, \dots ,$	$\bullet ,\overbrace{\circ , \circ , \circ , [P_n]}^{6},$	
$L^{f'}:$	$\bullet ,\overbrace{\circ , \circ , \circ , \circ ,[N_1]}^{7},$	$\bullet ,\overbrace{\circ ,\circ , \circ , \circ , [N_2]}^{{7}}, \dots ,$	$\bullet ,\overbrace{\circ , \circ , \circ , \circ ,[N_n]}^{7},$	

Recall that in a preference list $L^x$, all ‘$\bullet$’ symbols stand for items in $I_x$ (assigned by $\pi$ to *x*), while ‘$\circ$’ symbols stand for items in $D \setminus I_x$ (assigned by $\pi$ to some other agent in $\{v,v',w,w'\} \setminus \{x\}$). The numbers above braces indicate the length of the given series of items

Notice that we need to make sure that we do not “run out of” the necessary items when using ‘$\circ$’ symbols: for any agent *x*, the number of available dummy items is $|D \setminus I_x|\ge 12n$, while $L^x$ contains at most 12*n* dummies. Hence, ${\mathcal {P}}$ is indeed a profile.

The reduction presented can clearly be computed in polynomial time, so let us prove its correctness. First assume that *S* is a solution for $({\mathcal {P}},\pi )$ of size at most *k*. Note that $\pi$ assigns exactly 4*n* items to each agent except for *f* and $f'$, while it assigns 6*n* and 5*n* items to *f* and $f'$, respectively (recall that *f* receives all 3*n* items in ${\hat{U}}$, while $f'$ receives all 3*n* items in *U*). By $k=3n$, we get that *S* contains exactly 2*n* items of ${\hat{U}} \cup I_f$ and *n* items of $U \cup I_{f'}$. In particular, *S* does not contain any (dummy) item from *D*.

Fix some agent $x \in N \setminus \{f,f'\}$, and consider the first 7*j* items in the preference list of *x* for some $j \in \{1, \dots , 3n\}$. Observe that $L^x[1:7j]$ contains only *j* items assigned to *x* by $\pi$. However, $\pi$ cannot be proportional as long as the set consisting of the first $6j+1$ items in *x*’s preference list contains at most *j* items assigned to *x* by $\pi$. Hence we know that *S* must contain at least *j* items from $L^x[1:7j]$. Let $Q^x_j$ denote the set of items in $L^x[7j-6:7j]$ that are not in *D* and hence may be included in the solution *S*; e.g., $Q^v_1=\{a_1,{\hat{a}}_1\}$, $Q^v_2=\{b_1,{\hat{b}}_1\}$, and so on. Then our observation for each $x \in N\setminus \{f,f'\}$ can be written as

5 $$\begin{aligned} |S \cap (Q^x_1 \cup \dots \cup Q^x_j)| \ge j. \end{aligned}$$

However, notice that $Q^v_j=Q^{v'}_{3n+1-j}$ and also $Q^w_j=Q^{w'}_{3n+1-j}$, which implies

6 $$\begin{aligned} |S \cap (Q^x_{j+1} \cup \dots \cup Q^x_{3n})| =|S \cap (Q^{x'}_{3n-j} \cup \dots \cup Q^{x'}_{1})| \ge 3n-j \end{aligned}$$

for any $x \in N\setminus \{f,f'\}$ where we abuse notation by setting $v''=v$ and $w''=w$; note that we used Inequality (5) for agent $x'$ and index $3n-j$. Using that $|S|=3n$ and that the sets $Q^x_j$, $j \in \{1,\dots ,3n\}$, are mutually disjoint, it is straightforward to verify that Inequalities (5) and (6) can only hold for each value of $j \in \{1,\dots , 3n\}$ if

7 $$\begin{aligned} |S \cap Q^x_j|=1 \text { for every }j \in \{1,\dots , 3n\}. \end{aligned}$$

For agents *v* and $v'$, (7) implies that $u \in S$ if and only if ${\hat{u}} \notin S$ for any item $u \in U$. Taking into account the statement of (7) for agents *w* and $w'$, we obtain that either $V_i \subseteq S$ and ${\hat{V}}_i \cap S=\emptyset$, or ${\hat{V}}_i \subseteq S$ and $V_i \cap S=\emptyset$ for each $i \in \{1,\dots , n\}$.

Let us define a truth assignment $\alpha$ by setting variable $v_i \in V$ in $\varphi$ to true if and only if $V_i \subseteq S$. We claim that $\alpha$ is valid for $\varphi$, i.e., each clause in ${\mathcal {C}}$ contains exactly one variable $v_i$ for which $V_i \subseteq S$.

Considering the first 7*j* items from the preference list of *f* for some $j\in \{1,\dots , n\}$ and arguing similarly as before, we obtain that

8 $$\begin{aligned} |S \cap (P_1 \cup \dots \cup P_j)| \ge j. \end{aligned}$$

Analogously, from the first 8*j* items from the preference list of $f'$ (among which only *j* are assigned to $f'$ by $\pi$), we get

9 $$\begin{aligned} |S \cap (N_1 \cup \dots \cup N_j)| \ge 2j. \end{aligned}$$

However, notice that $|S \cap (P_j \cup N_j)|=3$ for any $j \in \{1,\dots ,n\}$. Hence, Inequalities (8) and (9) imply $|S \cap P_j|=1$ and $|S \cap N_j|=2$ for every $j \in \{1,\dots ,n\}$. This means exactly that $\alpha$ is valid for $\varphi$.

For the other direction, assume that there exists a valid truth assignment $\alpha$ for $\varphi$, and let $T_{\alpha }$ denote the set of true variables. First observe that $|T_{\alpha }|=n/3$ must hold, because each variable appears in exactly three clauses, and there are *n* clauses. We define a solution *S* for $({\mathcal {P}},\pi )$ by putting $a_i$, $b_i$, and $c_i$ into *S* for each $v_i \in T_{\alpha }$, and putting ${\hat{a}}_i$, ${\hat{b}}_i$, and ${\hat{c}}_i$ into *S* for each $v_i \in V \setminus T_{\alpha }$. Hence $|S\cap U|=n$ and $|S \cap {\hat{U}}|=2n$. Note that Inequality (5) holds for *S* for any agent $x \in N \setminus \{f,f'\}$ and index $j \in \{1,\dots ,3n\}$. Inequalities (8) and (9) hold as well for any $j \in \{1,\dots ,n\}$, due to the validity of $\alpha$. Based on these facts, it is easy to check that *S* is indeed a solution for $({\mathcal {P}},\pi )$. $\square$

Our next theorem shows that PID with Fixed Allocation remains computationally intractable even if the number of deletions allowed is small.

### Theorem 10

PID with Fixed Allocation *is* ${\mathsf {W}}[2]$-*hard when parameterized by the size* *k* *of the desired solution*.

### Proof

We are going to present an FPT-reduction from the Red-Blue Dominating Set problem. The input of this problem is a bipartite graph $G = (V, E)$ and an integer *k*, with *V* partitioned into a set $V_{red }=\{r_1,\dots , r_s\}$ of ‘red’ and a set $V_{blue }=\{b_1, \dots , b_t\}$ of ‘blue’ vertices. We denote by *N*(*v*) the set of neighbors of some vertex $v \in V$. The task is to decide if *G* contains a set $D \subseteq V_{red }$ of at most *k* red vertices that dominates all blue vertices, i.e., such that $N(b_j) \cap D \ne \emptyset$ for each $b_j \in V_{blue }$. Red-Blue Dominating Set is known to be ${{\mathsf {N}}}{{\mathsf {P}}}$-complete and ${\mathsf {W}}[2]$-complete when parameterized by *k* [12].

Let us construct an instance $I =({\mathcal {P}},\pi ,k)$ of PID with Fixed Allocation. We let ${\mathcal {P}}= (N,I,L)$, and we define the set of agents as $N=\{{\hat{r}}\} \cup {\hat{B}} \cup {\hat{D}}$ where ${\hat{B}}=\{{\hat{b}}_1, \dots , {\hat{b}}_t\}$, and ${\hat{D}}=\{{\hat{d}}_1, \dots , {\hat{d}}_{\varDelta }\}$ contains $\varDelta$ dummy agents for the smallest integer $\varDelta$ such that $\varDelta (s-k) \ge |N|=\varDelta +t+1$. We define the set of items as $I=V_{red } \cup B \cup D$ where $B=\{b^i_1, \dots , b^i_t \mid 1 \le i \le s-k\}$ contains $s-k$ copies of each blue vertex, and $D=\{d^i_1, \dots , d^i_\varDelta \mid 1 \le i \le s-k \}$ contains $s-k$ dummy items for each dummy agent. The allocation $\pi$ assigns every item of $V_{red }$ to agent ${\hat{r}}$, while each agent ${\hat{b}}_j \in {\hat{B}}$ is assigned items $b^1_j, \dots , b^{s-k}_j$, and each agent ${\hat{d}}_j \in {\hat{D}}$ is assigned items $d^1_j, \dots , d^{s-k}_j$.

To define the preferences of agents, we fix an arbitrary ordering $\prec$ over *I* so that we can omit listing all irrelevant items in the preference lists: the symbol ‘’ at the end of a preference list stands for the sequence of all remaining items according to their order in $\prec$. Also, we write [*X*] for a set *X* of items to denote their sequence according to $\prec$. Moreover, a series of ‘$\circ$’ symbols of length $\ell$ denotes the sequence of the first $\ell$ dummy items from *D* according to $\prec$. Now, the preference lists are as follows (allocation $\pi$ is indicated by underlining the items assigned to the given agent *x* in $L^x$).Note that the preference list of an agent ${\hat{b}}_j \in {\hat{B}}$ contains at most |*N*| dummy items, whose existence is ensured by our choice of $\varDelta$.

The presented reduction is clearly a polynomial-time reduction as well as a parameterized one (with *k* being the parameter in both instances). To prove its correctness, let us first assume that *S* is a solution of size at most *k* for $({\mathcal {P}},\pi )$. Note that $\pi$ assigns $s-k$ items to each agent, except for agent ${\hat{r}}$ who gets *s* items. Hence, $S \subseteq \pi ^{-1}({\hat{r}})=V_{red }$ and $|S|=k$ follows. We claim that *S* dominates all blue vertices in *G*: indeed, if $S \cap N(b_j) =\emptyset$ for some $b_j \in V_{blue }$ in *G*, then agent ${\hat{b}}_j$ becomes envious at index $|N|+1$ in ${\mathcal {P}}-S$, since $L^{{\hat{b}}_j}[1:|N|+1]$ contains only one item assigned to ${\hat{b}}_j$ by $\pi$, a contradiction.

For the other direction, it is straightforward to verify that any set *S* of *k* red vertices that dominates all blue vertices in *G* yields a solution to $({\mathcal {P}},\pi )$ as well, because if no agent of ${\hat{B}}$ becomes envious at index $|N|+1$ in ${\mathcal {P}}-S$, then $\pi$ is guaranteed to be proportional in ${\mathcal {P}}-S$. $\square$

## Conclusion and Open Questions

In Section 4 we have shown that Proportionality by Item Deletion is polynomial-time solvable if there are only three agents. In comparison, the problem of obtaining an envy-free allocation by item deletion was shown to be polynomial-time solvable for two agents, but becomes NP-hard already for three agents [4]. We have also proved that if the number of agents is unbounded, then PID becomes NP-hard, and practically intractable already when we want to delete only a small number of items, as shown by the $\mathsf {W[3]}$-hardness result of Theorem 2.

The complexity of PID remains open for the case when the number of agents is a constant greater than 3. Is it true that for any constant *n*, there exists a polynomial-time algorithm that solves PID in polynomial time for *n* agents? The reason why our algorithm is not directly applicable for more than three agents is that Lemma 4 relies heavily on the properties of minimal obstructions observed in Lemma 2; these properties imply a very strict structure for two properly intersecting minimal obstructions. For the case of more than three agents, there are more possibilities how minimal obstructions may properly intersect, and so we cannot obtain a variant of Lemma 4 for $n\ge 4$: it is an open question whether for $n \ge 4$ it holds that any inclusion-wise minimal solution *S* contains at most $n-1$ items from a minimal obstruction ${\mathcal {Q}}$. In fact, it is not even known whether there is a bound *f*(*n*) on the number of items in $S \cap I({\mathcal {Q}})$ for some function *f*; we believe that establishing such a bound would imply the existence of a polynomial-time algorithm for PID for any constant *n*.

Supposing that there does exist a polynomial-time algorithm for PID for any constant *n*, can we find an FPT-algorithm with respect to the parameter *n*? If not—that is, if PID turns out to be ${{\mathsf {N}}}{{\mathsf {P}}}$-hard for some constant number of agents—then can we at least give an FPT-algorithm with parameter *k* for a constant number of agents (or maybe with combined parameter (*k*, *n*))?

Since approximation seems hopeless w.r.t. the number of deletions, it may be interesting to see whether there might exist an approximation w.r.t. the number of items obtained by each agent in a proportional way. Our results from Corollary 1 and Theorem 1 show that we cannot get a polynomial-time approximation with a ratio better than 2. Is this lower bound sharp?

Regarding PID with Fixed Allocation, we gave a polynomial-time algorithm for $n=2$, but proved the problem to be ${{\mathsf {N}}}{{\mathsf {P}}}$-complete for $n=6$. This leaves open the case when the number of agents is in $\{3,4,5\}$; it would be interesting to close the gap.

Finally, there is ample space for future research if we consider different control actions (such as adding or replacing items), different notions of fairness, or different models for agents’ preferences.

## Figures and Tables

**Fig. 7:**
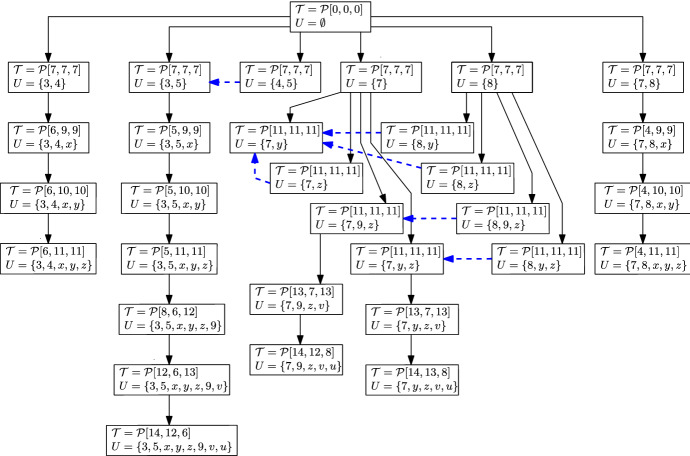
Illustration for the search tree traversed by algorithm $$Extend $$ on the example of Appendix A. Black arrows indicate a recursive call, while blue dashed arrows indicate strong equivalence (pointing towards the earlier call)

## Appendix A Example for Running Algorithm $$Extend $$

In this section we show how the algorithm runs on a profile ${\mathcal {P}}$ with agents *a*, *b*, and *c* ranking the item set $\{1,2, \dots , 9, x,y,z,v,u\}$ as below:

$$\begin{aligned}{}\begin{array}[t]{rl} \hbox {Profile }{\mathcal {P}}: \quad a: &{} 1, \ 2, 3, 6, \ 4, 7, 8, \ 9, y, x, \ z, v, 5, \ u. \\ b: &{} 2, \ 1, 5, 3, \ 4, 8, 6, \ x, 7, y, \ z, u, 9, \ v. \\ c: &{} 3, \ 4, 6, 2, \ 5, 7, 1, \ x, 8, y, \ z, 9, v, \ u. \end{array} \end{aligned}$$

At each step, we will only indicate Step 0, Step 1, or Step 3 if it leads to an output. If not, then we give the minimal obstruction *Q* and the branching set ${\mathcal {Y}}$ computed in Steps 2 and 4. The numbering of the calls reflects how the algorithm performs the recursive calls in Step 5; Fig. 7 depicts the search tree traversed by the algorithm. Table 2 shows the content of SolTable. Sometimes, in order to omit details and avoid tedium, we will shorten the description of a call.

**Table 2:** The contents of SolTable as a result of running $Extend ({\mathcal {T}}_{\emptyset },\emptyset )$ on the example of Appendix A. The entries appear according to the order in which the algorithm stores them

Prefix	Partial solution	Extension
${\mathcal {P}}[6,11,11]$	$\{3,4,x,y,z\}$	$\emptyset$
${\mathcal {P}}[6,10,10]$	$\{3,4,x,y\}$	$\{z\}$
${\mathcal {P}}[6,9,9]$	$\{3,4,x\}$	$\{y,z\}$
${\mathcal {P}}[7,7,7]$	$\{3,4\}$	$\{x,y,z\}$
${\mathcal {P}}[14,12,6]$	$\{3,5,x,y,z,9,v,u\}$	$\emptyset$
${\mathcal {P}}[12,6,13]$	$\{3,5,x,y,z,9,v\}$	$\{u\}$
${\mathcal {P}}[8,6,12]$	$\{3,5,x,y,z,9\}$	$\{v,u\}$
${\mathcal {P}}[5,11,11]$	$\{3,5,x,y,z\}$	$\{9,v,u\}$
${\mathcal {P}}[5,10,10]$	$\{3,5,x,y\}$	$\{z,9,v,u\}$
${\mathcal {P}}[5,9,9]$	$\{3,5,x\}$	$\{y,z,9,v,u\}$
${\mathcal {P}}[7,7,7]$	$\{3,5\}$	$\{x,y,z,9,v,u\}$
${\mathcal {P}}[11,11,11]$	$\{7,y\}$	$\emptyset$
${\mathcal {P}}[14,12,8]$	$\{7,9,z,v,u\}$	$\emptyset$
${\mathcal {P}}[13,7,13]$	$\{7,9,z,v\}$	$\{u\}$
${\mathcal {P}}[11,11,11]$	$\{7,9,z\}$	$\{v,u\}$
${\mathcal {P}}[14,13,8]$	$\{7,y,z,v,u\}$	$\emptyset$
${\mathcal {P}}[13,7,13]$	$\{7,y,z,v\}$	$\{u\}$
${\mathcal {P}}[11,11,11]$	$\{7,y,z\}$	$\{v,u\}$
${\mathcal {P}}[7,7,7]$	$\{7\}$	$\{y\}$
${\mathcal {P}}[7,7,7]$	$\{8\}$	$\{y\}$
${\mathcal {P}}[4,11,11]$	$\{7,8,x,y,z,\}$	$\emptyset$
${\mathcal {P}}[4,10,10]$	$\{7,8,x,y\}$	$\{z\}$
${\mathcal {P}}[4,9,9]$	$\{7,8,x\}$	$\{y,z\}$
${\mathcal {P}}[7,7,7]$	$\{7,8\}$	$\{x,y,z\}$
${\mathcal {P}}[0,0,0]$	$\emptyset$	$\{7,y\}$

## Footnotes

1 For a complete proof of the correctness of their algorithm, see also the work by Aziz et al. [4].

2 Here, we use an effective variant of “little o” (see, e.g. Flum and Grohe [15, Definition 3.22]).

3 Seemingly it may be incorrect to say that ${\mathcal {R}}$ is *contained* in ${\mathcal {Q}}$ because ${\mathcal {R}}$ is a prefix of ${\mathcal {P}}'$ while ${\mathcal {Q}}$ is a prefix of ${\mathcal {P}}$; however, recall that the definition of containment only depends on the notion of size.

4 If ${\mathcal {T}}_1 -U_0={\mathcal {T}}_2-U_0={\mathcal {Q}}$, then $({\mathcal {T}}_1 \cup T_2)-U_0={\mathcal {Q}}$ also holds (where the union of ${\mathcal {T}}_1$ and ${\mathcal {T}}_2$ is the unique smallest prefix containing both); hence, ${\mathcal {T}}$ is well defined.

## References

[1] Arora S, Barak B (2009). Computational Complexity - A Modern Approach.

[2] Aziz, H., Bouveret, S., Caragiannis, I., Giagkousi, I., Lang, J.: Knowledge, fairness, and social constraints. In *Proceedings of the 32nd AAAI Conference on Artificial Intelligence (AAAI 2018)*, pages 4638–4645, New Orleans, Louisiana, United States, (2018)

[3] Aziz H, Gaspers S, Mackenzie S, Walsh T (2015). Fair assignment of indivisible objects under ordinal preferences. Artif. Intel..

[4] Aziz, H., Schlotter, I., Walsh, T.: Control of fair division. In *Proceedings of the 25th International Joint Conference on Artificial Intelligence (IJCAI 2016)*, pages 67–73, (2016)

[5] Bartholdi JJ, Tovey CA, Trick MA (1992). How hard is it to control an election?. Mathemat. Comp. Modelling.

[6] Bouveret, S., Chevaleyre, Y., Maudet, N.: Fair allocation of indivisible goods. In F. Brandt, V. Conitzer, U. Endriss, J. Lang, and A. D. Procaccia, editors, *Handbook of Computational Social Choice*, pages 284–310. Cambridge University Press, (2016)

[7] Brams SJ, Kilgour DM, Klamler C (2014). Two-person fair division of indivisible items: An efficient, envy-free algorithm. Notic. American Mathemat. Soc..

[8] Caragiannis, I., Gravin, N., Huang, X.: Envy-freeness up to any item with high nash welfare: The virtue of donating items. In *Proceedings of the 2019 ACM Conference on Economics and Computation (EC 2019)*, page 527–545, New York, NY, USA, 2019. ACM (2019)

[9] Chen J, Huang X, Kanj IA, Xia G (2006). Strong computational lower bounds via parameterized complexity. J. Comp. Sys. Sci..

[10] Chen, Y., Shah, N.: Ignorance is often bliss: Envy with incomplete information, 2017. Working paper (2017)

[11] Downey RG, Fellows MR (1995). Fixed-parameter tractability and completeness I: Basic results. SIAM J. Comp..

[12] Downey RG, Fellows MR (1999). Parameterized Complexity.

[13] Downey RG, Fellows MR (2013). Fundamentals of parameterized complexity.

[14] Flum J, Grohe M (2005). Model-checking problems as a basis for parameterized intractability. Logic. Methods . Comp. Sci..

[15] Flum, J., Grohe, M.: Parameterized Complexity Theory. Texts in Theoretical Computer Science, vol. XIV. An EATCS Series. Springer Verlag, Berlin (2006)

[16] Halpern, D., Shah, N.: Fair division with subsidy. In : D. Fotakis and E. Markakis, editors, *Proceedings of the 12th International Symposium on Algorithmic Game Theory (SAGT 2019)*, *Lecture Notes in Computer Science*, pp. 374–389. Springer, New york (2019)

[17] Hopcroft JE, Karp RM (1973). An $$n^{5/2}$$ algorithm for maximum matchings in bipartite graphs. SIAM Journal on Computing.

[18] Hosseini, H., Sikdar, S., Vaish, R., Wang, H., Xia, L.: Fair division through information withholding. In *The 34th AAAI Conference on Artificial Intelligence (AAAI 2020)*, pages 2014–2021. AAAI Press, (2020)

[19] Impagliazzo R, Paturi R, Zane F (2001). Which problems have strongly exponential complexity?. J. Comp. Sys. Sci..

[20] Lipton, R.J., Markakis, E., Mossel, E., Saberi, A.: On approximately fair allocations of indivisible goods. In *EC 2004: Proc. of the 5th ACM Conference on Electronic Commerce*, pages 125–131, (2004)

[21] Moore C, Robson JM (2001). Hard tiling problems with simple tiles. Discret. Comput. Geom..

[22] Nguyen, T., Vohra, R.: Near feasible stable matchings. In *Proceedings of the Sixteenth ACM Conference on Economics and Computation (EC 2015)*, pages 41–42, (2015)

[23] Pruhs, K.R., Woeginger, G.J.: Divorcing made easy. In E. Kranakis, D. Krizanc, and F. Luccio, editors, *Proceedings of the 6th International conference on Fun with Algorithms (FUN 2012)*, volume 7288 of *LNCS*, pages 305–314. Springer, (2012)

[24] Segal-Halevi, E., Hassidim, A., Aumann, Y.: Waste makes haste: Bounded time protocols for envy-free cake cutting with free disposal. In *AAMAS 2014: In Proc. of the 14th International Conference on Autonomous Agents and Multi-Agent Systems*, pages 901–908, (2015)

[25] Thulasiraman, K., Arumugam, S., Brandstädt, A., Nishizeki, T.: Handbook of Graph Theory, Combinatorial Optimization, and Algorithms. CRC Press, Chapman & Hall/CRC Computer and Information Science Series (2015)

